# Antibiotic Consumption Patterns in Three Romanian Hospitals: A Five-Year Retrospective Study

**DOI:** 10.3390/antibiotics15090901

**Published:** 2026-09-13

**Authors:** Radu-Daniel Duvlea, Robert Viorel Ancuceanu, Ana-Maria Ispas, Adriana-Elena Tăerel

**Affiliations:** 1 Department of Management and Pharmaceutical Marketing, Faculty of Pharmacy, “Carol Davila” University of Medicine and Pharmacy, 020956 Bucharest, Romania; 2 Department of Pharmaceutical Botany and Cell Biology, Faculty of Pharmacy, “Carol Davila” University of Medicine and Pharmacy, 020956 Bucharest, Romania

**Keywords:** antibiotic consumption, AWaRe classification, DDD/100 bed-days, hospital

## Abstract

**Background**: Monitoring antibiotic consumption represents an important objective for antimicrobial stewardship programs in hospitals. The aim of this study was to analyze antibiotic consumption patterns in three Romanian hospitals. **Methods:** A five-year retrospective study was conducted using monthly pharmacy consumption data from three Romanian hospitals for the years 2018, 2019, 2023, 2024, and 2025. Antibiotic consumption was quantified using defined daily doses (DDD) per 100 bed-days. Antibiotics were grouped using the World Health Organization AWaRe (Access, Watch, Reserve) classification. **Results**: The analysis revealed statistically significant differences in overall antibiotic consumption among the three hospitals (*p* < 0.001). A significant annual correlation between hospitalization days and antibiotic consumption was observed for some years in two of the facilities. Post hoc analysis confirmed statistically significant differences across all AWaRe category pairwise comparisons. Watch antibiotics had the highest consumption rates (over 60%). By therapeutic classes, the highest overall consumption was for cephalosporins, fluoroquinolones, penicillins, and carbapenems. Cefuroxime accounted for the highest cumulative consumption, followed by ceftriaxone, meropenem, vancomycin, ciprofloxacin, and metronidazole. **Conclusions**: Antibiotic consumption differed among the participating hospitals and study years. Monitoring antibiotic consumption provides valuable information on hospital antibiotic utilization patterns and can support evidence-based antimicrobial stewardship in hospitals.

## 1. Introduction

Antimicrobial resistance (AMR) represents one of the top global public health threats in the 21st century. In the World Health Organization (WHO) European Region, AMR is directly responsible for 133 000 deaths each year and indirectly linked to 541,000 deaths [1]. The rising incidence of multidrug-resistant (MDR) microbes is a safety concern for healthcare professionals, patients, and healthcare systems [2].

Hospitals represent one of the most important settings for antibiotic use because they provide care for patients with severe infections, multiple comorbidities, and complex medical or surgical conditions. Therefore, hospital programs aimed at improving antibiotic use, referred to as antimicrobial stewardship programs (ASPs), can optimize the treatment of infections while minimizing adverse antibiotic events, including the development and spread of AMR [3].

Monitoring antibiotic consumption provides essential information for evaluating prescribing practices, identifying changes in utilization patterns over time, and supporting quality improvement initiatives. Among available surveillance methodologies, the Anatomical Therapeutic Chemical/Defined Daily Dose (ATC/DDD) methodology developed by the WHO is the internationally accepted standard for measuring antibiotic utilization. Expressing antibiotic consumption as defined daily doses (DDD) per 100 bed-days enables the standardization of medicine groups and provides a stable metric to compare drug use between countries, regions, and different healthcare settings [4].

To further support antimicrobial stewardship, the WHO introduced the AWaRe (Access, Watch, Reserve) classification in 2017, as a tool to monitor antibiotic use, define targets, and evaluate the impact of stewardship interventions aimed at optimizing treatment and curbing antibiotic resistance [5]. The AWaRe framework encourages the preferential use of Access antibiotics whenever clinically appropriate while preserving Watch and Reserve antibiotics for situations in which they are clearly indicated [6]. Thus, combining quantitative ATC/DDD consumption data with the AWaRe classification has become an important tool for assessing prescribing patterns and supporting antimicrobial stewardship programs in hospitals.

Romania has reported high levels of antimicrobial consumption and antimicrobial resistance, with a total antimicrobial consumption rate of 27.4 DDD per 1000 inhabitants per day and a broad-spectrum hospital proportion of 60.1%, representing the second-highest values in a study of 26 European Union/European Economic Area (EU/EEA) countries [7].

Analyses of antibiotic consumption patterns have gained interest in recent years [8,9,10,11]. While several studies have focused on the immediate impact of the COVID-19 pandemic on hospital prescribing practices by comparing pre-pandemic and pandemic periods [12,13,14,15,16,17,18,19,20], evaluations covering more recent years remain scarce. Hospital-level data describing antibiotic consumption during more recent years remain limited, particularly in Romania. Standardized multicenter surveillance may provide valuable information on differences in antibiotic utilization across hospitals and study years and may support antimicrobial stewardship activities.

Therefore, this study aimed to evaluate antibiotic consumption across three Romanian hospitals in 2018, 2019, 2023, 2024, and 2025. The main objectives were to compare antibiotic consumption across hospitals and study years; assess the relationship between antibiotic consumption and hospital bed-days; and characterize antibiotic utilization according to the WHO AWaRe classification, therapeutic classes, individual antibacterial agents, and route of administration.

## 2. Results

### 2.1. Overall Antibiotic Consumption

**Figure 1:**
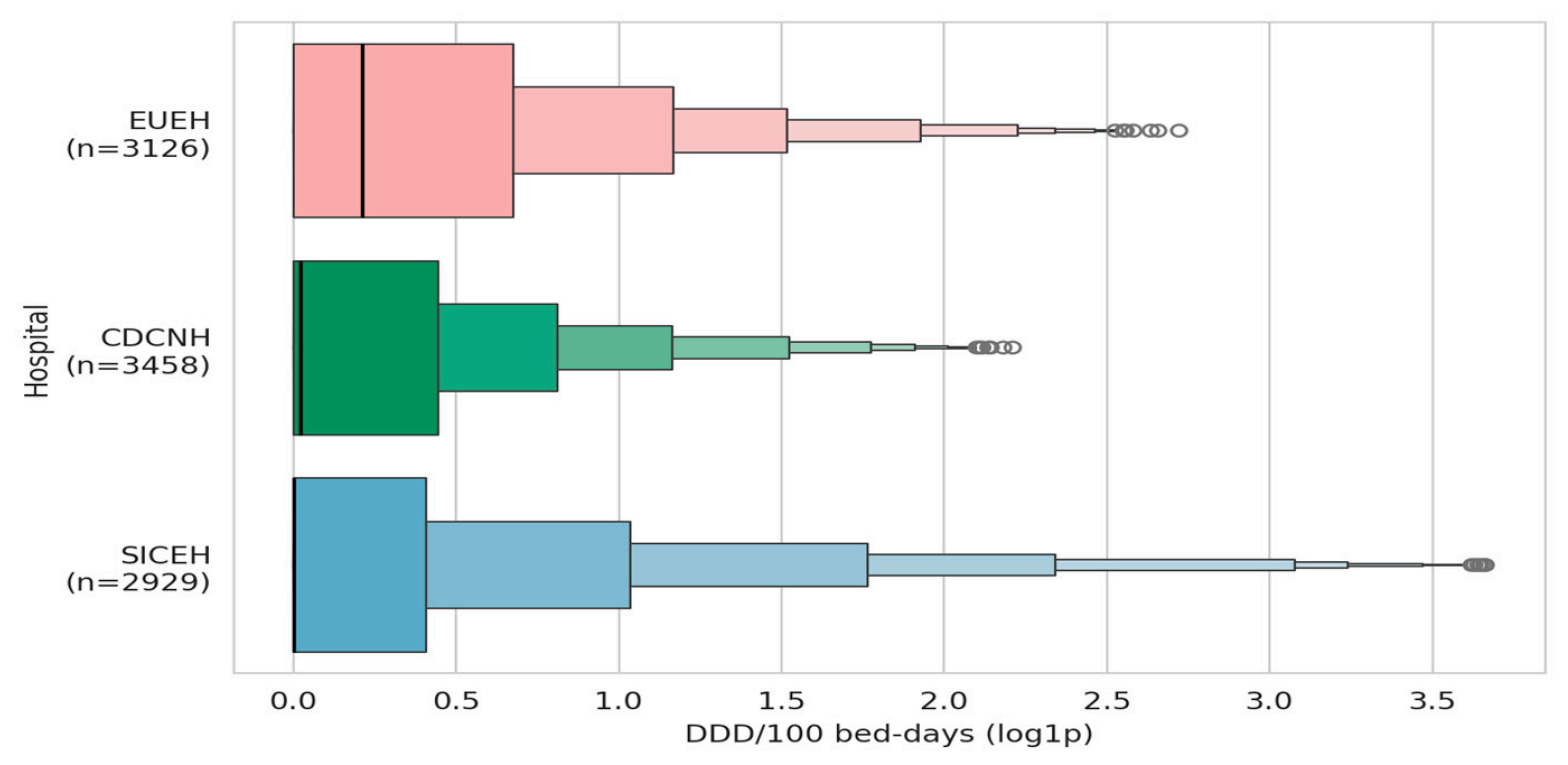
The distribution of overall antibiotic consumption by hospital. Boxes show successively narrower quantile ranges (letter-value/boxen plot display); the center line represents the median, and individual points denote extreme values beyond the plotted quantiles. EUEH = Elias University Emergency Hospital; CDCNH = Dr. Carol Davila Clinical Nephrology Hospital; SICEH = Sf. Ioan Clinical Emergency Hospital; DDD = Daily Defined Dose.

A comparative analysis of the antibiotic consumption in the three studied hospitals was presented in Figure 1. The analysis revealed statistically significant differences among hospitals (Kruskal–Wallis test, *p* < 0.001). Post hoc analysis using the Dunn test, with Bonferroni correction of *p*-values for multiple comparisons, showed significant differences between Elias Hospital and Nephrology Hospital (adjusted *p* < 0.001). Differences were also observed between Elias Hospital and Sf. Ioan Hospital (adjusted *p* < 0.001). In contrast, the difference between Sf. Ioan Hospital and Nephrology Hospital was not statistically significant after applying the correction for multiple comparisons (adjusted *p* = 0.078). Median antibiotic consumption was 0.234 DDD/100 bed-days at EUEH, 0.000 DDD/100 bed-days at SICEH, and 0.024 DDD/100 bed-days at CDCNH. Effect sizes were also calculated in addition to *p*-values because the comparisons involved a large number of antibiotic-month observations (*n* = 9513). The omnibus Kruskal–Wallis effect size was modest (epsilon-squared = 0.037), and pairwise rank-biserial correlations were r = −0.22 (95% CI [−0.24, −0.19]) for Elias Hospital versus Nephrology Hospital, r = −0.24 (95% CI [−0.27, −0.22]) for Elias versus Sf. Ioan Hospital, and r = −0.03 (95% CI [−0.06, −0.01]) for Sf. Ioan versus Nephrology Hospital. The results indicate that the overall difference identified between the hospitals is primarily driven by the distinct pattern of antibiotic use observed at Elias Hospital.

**Figure 2:**
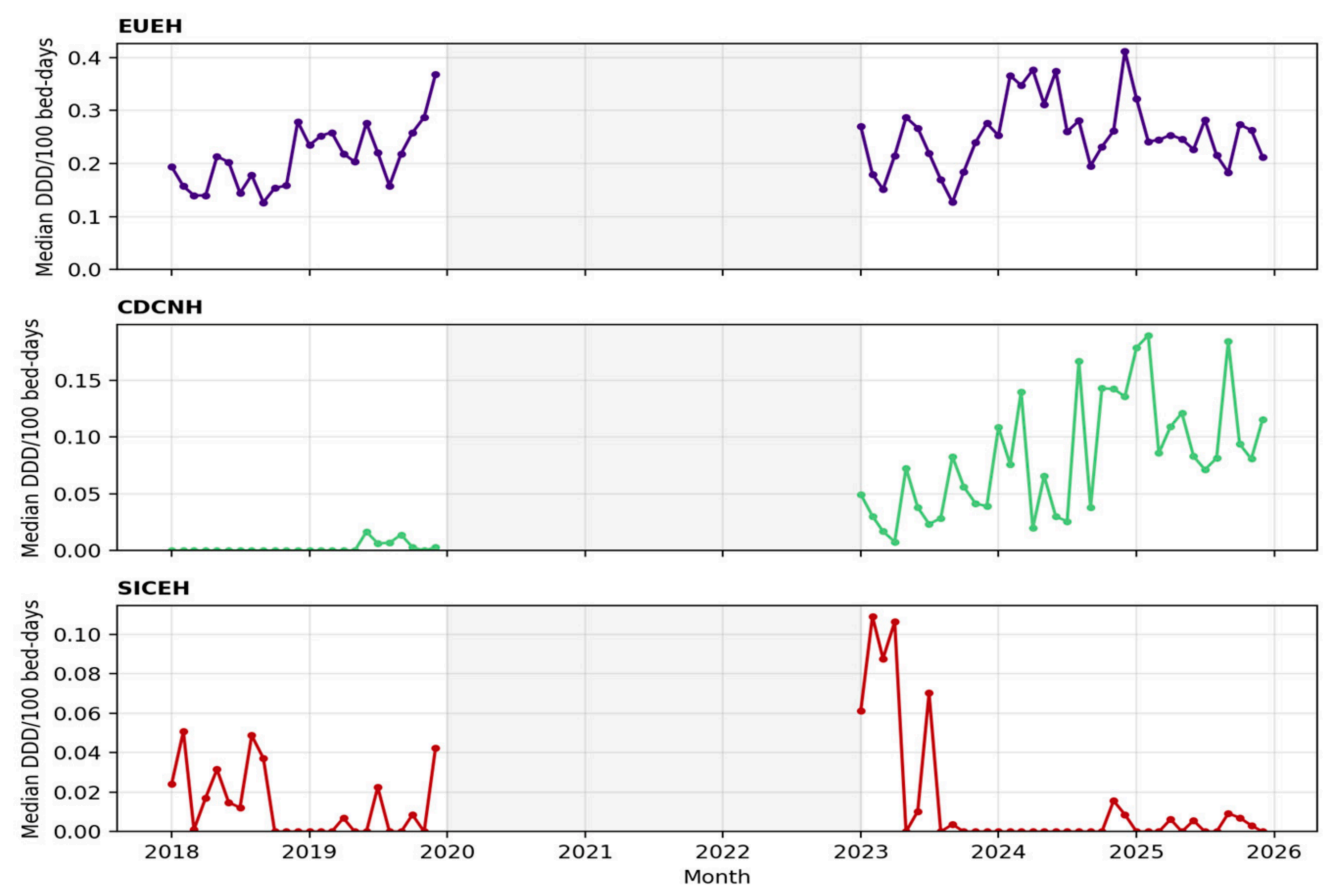
Monthly median antibiotic consumption (DDD/100 bed-days) by hospital across the five study years. Each hospital is shown on its own *y*-axis scale; the shaded band marks the period 2020–2022, for which data were unavailable. EUEH = Elias University Emergency Hospital; CDCNH = Dr. Carol Davila Clinical Nephrology Hospital; SICEH = Sf. Ioan Clinical Emergency Hospital.

Monthly median antibiotic consumption varied across the three hospitals and study years, as illustrated in Figure 2.

Nephrology Hospital showed very low median DDD/100 bed-day values during 2018–2019, while during the 2023–2025 period there was an increase in consumption levels. The lowest median values were observed for Sf. Ioan Hospital, with episodes of increase during certain months, particularly at the beginning of the 2023–2025 period. The break between 2020 and 2022 is shown in Figure 2 to avoid implying continuity in the absence of data. There were no statistically significant variations (Kruskal–Wallis: H(11) = 2.06; *p* = 0.998) in the distribution of DDD/100 bed-days across the months of the year when all observations were analyzed together (Figure S1).

Annual correlation estimates were based on 12 monthly observations per hospital and should therefore be interpreted cautiously.

**Figure 3:**
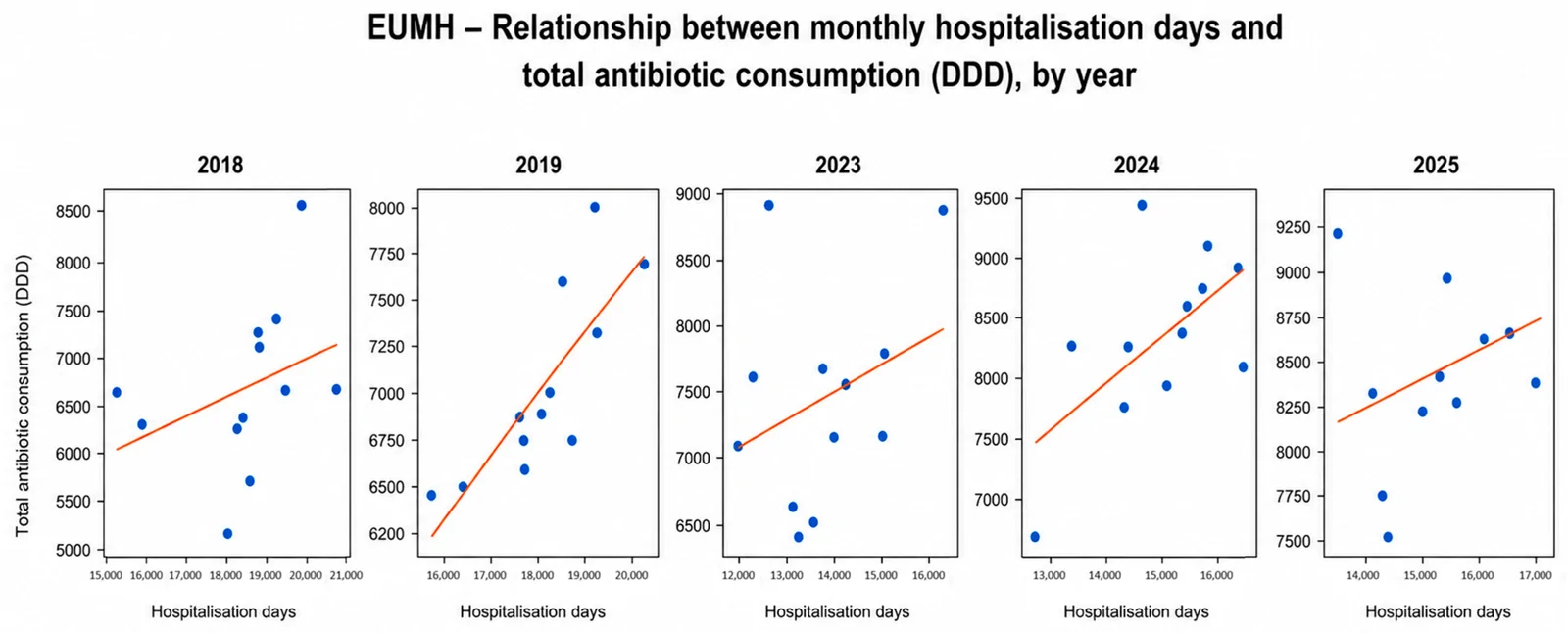
Correlation between monthly hospitalization days and total antibiotic consumption at Elias Hospital by years.

A significant annual correlation was observed at Elias Hospital between hospitalization days and antibiotic consumption in 2019 (r = 0.81, *p* = 0.001) and 2024 (r = 0.61, *p* = 0.034) (95% CI, Fisher z: 2019 [0.44, 0.94]; 2024 [0.06, 0.88]) (Figure 3).

For Nephrology Hospital, positive and significant correlations were identified in 2018, 2019, and 2023 (r = 0.79–0.83, *p* < 0.01), with a significant positive correlation also observed in 2025 (r = 0.67, *p* = 0.016).

**Figure 4:**
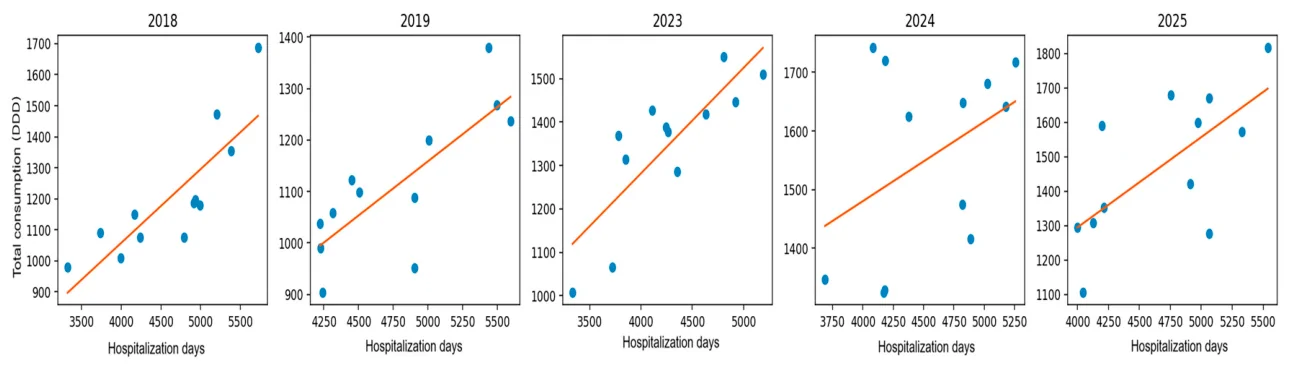
Monthly correlation dynamics between monthly hospitalization days and total antibiotic consumption at Nephrology Hospital by years.

The correlation for 2024 was not significant (Figure 4). The corresponding 95% confidence intervals for 2018, 2019, and 2023 ranged from a lower bound of 0.40–0.49 to an upper bound of 0.94–0.95 and from 0.16 to 0.90 for the 2025 correlation. In 2024, the confidence interval crossed zero ([−0.21, 0.80]), consistent with the non-significant result.

**Figure 5:**
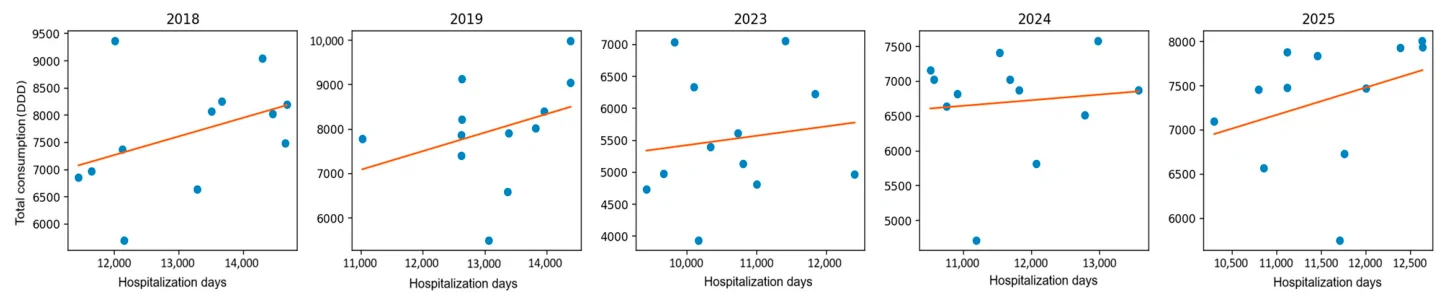
Correlation between monthly hospitalization days and total antibiotic consumption at Sf. Ioan Hospital by years.

Regarding Sf. Ioan Hospital, no significant annual correlation was observed between hospitalization days and antibiotic consumption (r = 0.10–0.40, *p* > 0.05) (Figure 5). In all five years, the 95% confidence interval for r crossed zero, consistent with the absence of a significant association.

An analysis of the correlation between hospital bed-days and consumption over the five-year study period (*n* = 60 months) for each hospital showed a significant association at Sf. Ioan Hospital (r = 0.59, *p* < 0.001) and Nephrology Hospital (r = 0.43, *p* < 0.001), whereas no significant correlation was observed at Elias Hospital (r = −0.23, *p* = 0.077; rho = −0.19, *p* = 0.154). The 95% confidence intervals (Fisher z) were [0.39, 0.73] for Sf. Ioan, [0.20, 0.62] for Nephrology Hospital, and [−0.46, 0.03] for Elias Hospital, the latter including zero, consistent with the non-significant result.

### 2.2. Antibiotic Consumption Based on the AWaRe Classification

**Figure 6:**
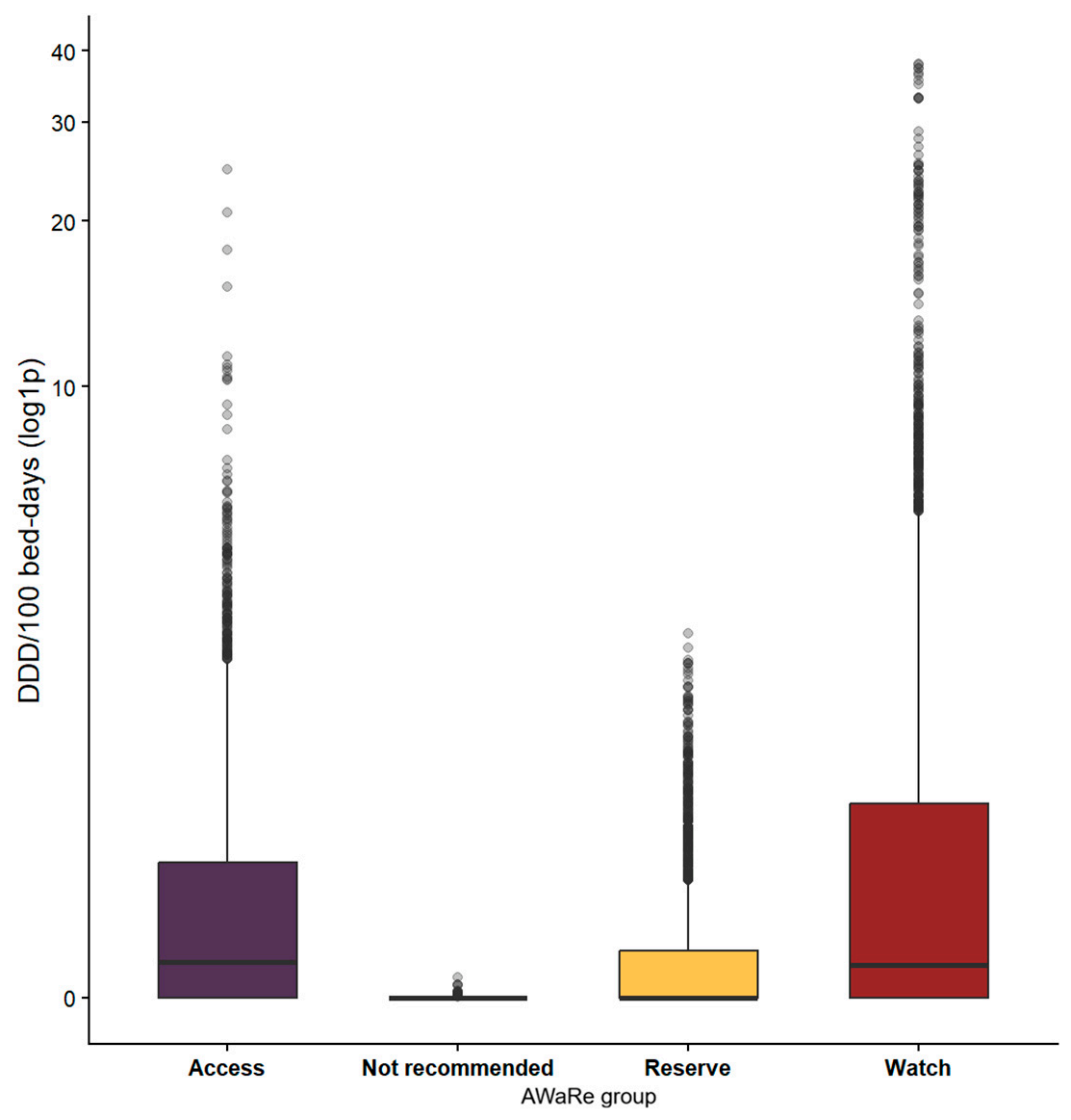
Distribution of overall antibiotic consumption by WHO AWaRe classification.

A comparative analysis of the distribution of antibiotic use according to WHO AWaRe classification is presented in Figure 6. Statistically significant differences were observed among the four categories analyzed (Kruskal–Wallis test; *p* < 0.001). The corresponding omnibus effect size was ε^2^ = 0.075, indicating a modest difference among AWaRe categories. Post hoc analysis, performed using the Dunn test with Bonferroni correction of *p*-values for multiple comparisons, revealed statistically significant differences between all pairs of AWaRe categories.

Highly significant differences were identified between Access and Not Recommended, Access and Reserve, Not Recommended and Reserve, Not Recommended and Watch, and Reserve and Watch (all adjusted *p*-values < 0.001). The difference between the Access and Watch categories was also statistically significant, though less pronounced (adjusted *p* = 0.031). For the Access–Watch comparison, the rank-biserial correlation was r = −0.02 (95% CI [−0.05, 0.00]), indicating a negligible effect despite statistical significance. This finding showed that statistical significance at this sample size does not necessarily imply a significant difference in magnitude (Table S1).

Table 1 presents the distribution of overall antibiotic consumption according to the WHO AWaRe classification by hospital and study year.

The analysis of each AWaRe group across study years showed statistically significant differences in all three hospitals. Only statistically significant pairwise post hoc comparisons of AWaRe antibiotic consumption across study years for each hospital are presented in the Supplementary Materials (Table S2).

**Figure 7:**
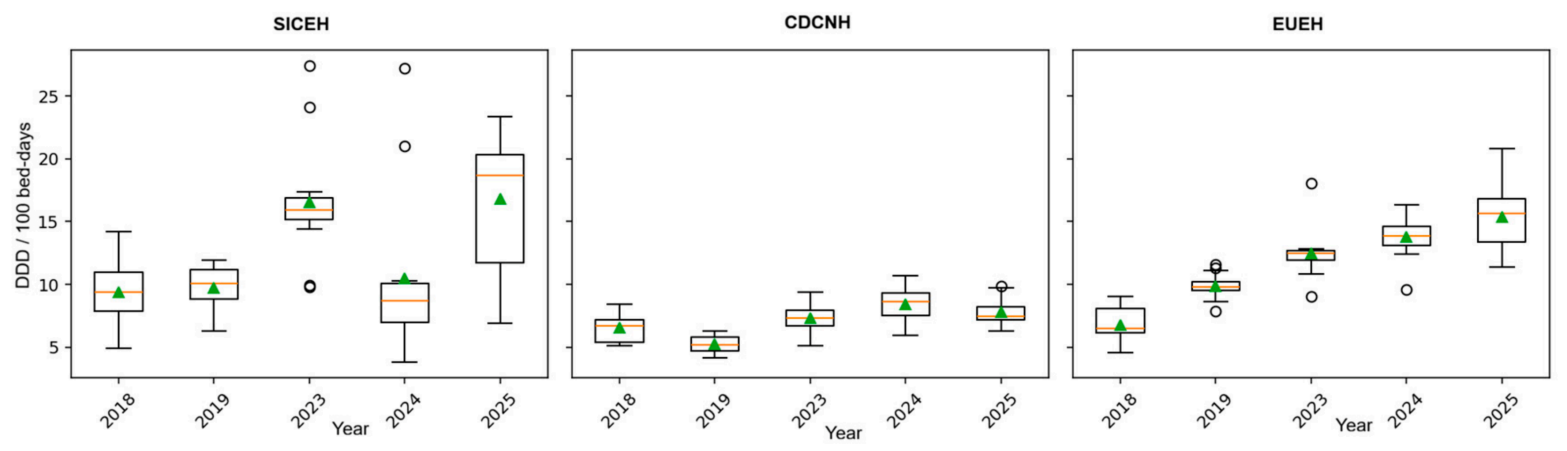
The distribution of the monthly Access group of antibiotic consumption by year and hospital. Boxplots show the median (orange line), mean (green triangle), interquartile range (box), and outliers (open circles).

At Sf. Ioan Hospital, the Access group showed significant differences when comparing 2018 and 2019 with 2023 and 2025. At Nephrology Hospital, significant differences in Access group usage were noted between 2018 and 2019, between 2018 and 2024, and also between 2019 and 2023 and 2024 and 2025. At Elias Hospital, significant differences for Access antibiotic consumption were observed across all years, except for 2024 versus 2025 (Figure 7).

**Figure 8:**
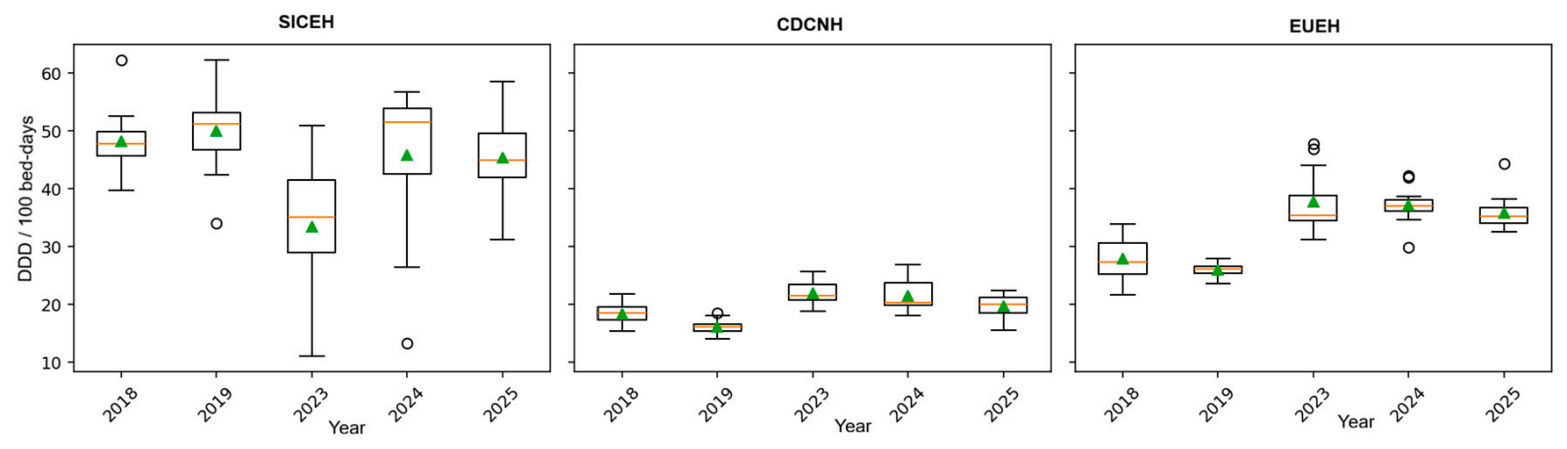
The distribution of the monthly Watch group of antibiotic consumption by year and hospital. Boxplots show the median (orange line), mean (green triangle), interquartile range (box), and outliers (open circles).

Regarding the Watch antibiotics, significant differences were observed at Sf. Ioan Hospital when comparing 2018 to 2023 and 2019 to 2023. At Nephrology Hospital, significant differences were noted between 2018 and 2019 versus 2023, 2024, and 2025, with the exception of 2018 versus 2025. At Elias Hospital, differences in Watch group consumption were observed between 2018 and 2019 and the later study years (2023, 2024, and 2025) (Figure 8).

**Figure 9:**
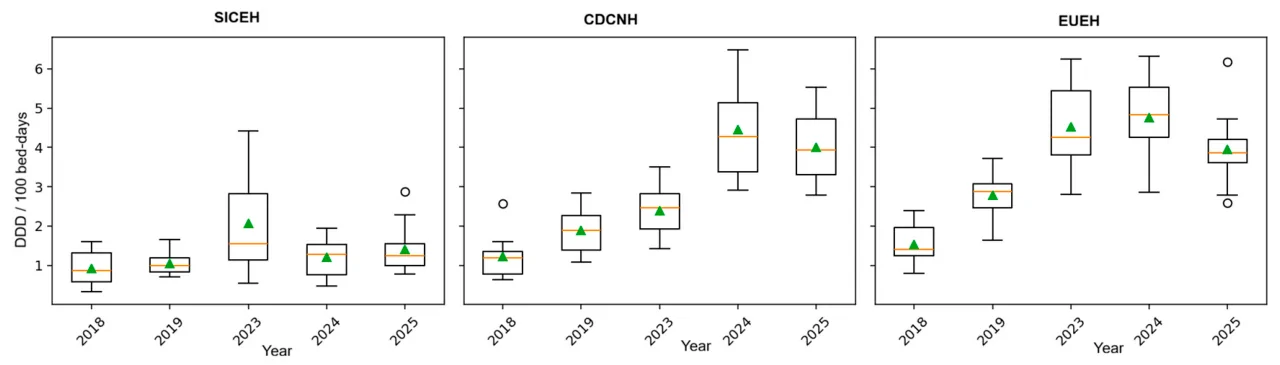
The distribution of the monthly Reserve group of antibiotic consumption by year and hospital. Boxplots show the median (orange line), mean (green triangle), interquartile range (box), and outliers (open circles).

**Figure 10:**
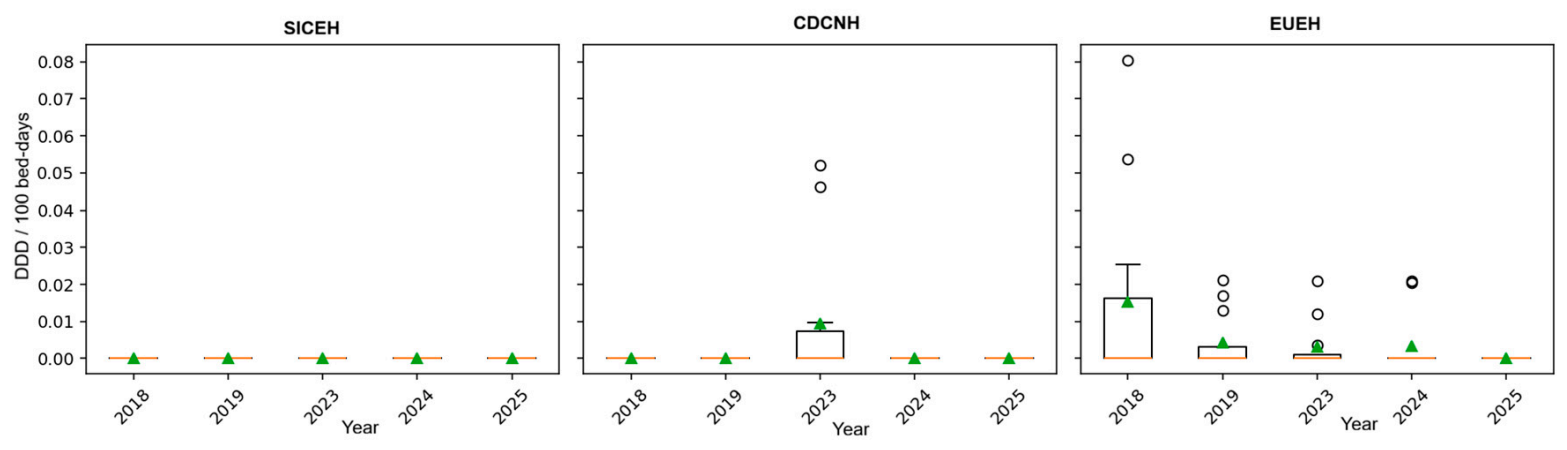
The distribution of the monthly Not Recommended group of antibiotic consumption by year and hospital. Boxplots show the median (orange line), mean (green triangle), interquartile range (box), and outliers (open circles).

No significant differences were observed for the Reserve group at Sf. Ioan Hospital (*p* = 0.499) and the Not Recommended group at Elias (*p* = 0.14). For the Reserve group, significant differences were identified at Nephrology Hospital between 2018 and 2023, 2024, and 2025; between 2019 and 2024 and 2025; and between 2023 and 2024 and 2025. At Elias Hospital, significant differences for the Reserve agents were observed across study years (Figure 9 and Figure 10).

In each hospital and study year, statistically significant differences were observed among the four AWaRe groups (*p* < 0.001). Watch and Access antibiotics accounted for the largest proportions of antibiotic consumption, whereas Reserve and Not Recommended antibiotics represented substantially smaller proportions. The post hoc analysis showed that all pairs differed (adjusted *p*-value < 0.05), as illustrated in Supplementary Materials (Figures S2–S4).

### 2.3. Analysis by Classes and Active Substances

**Figure 11:**
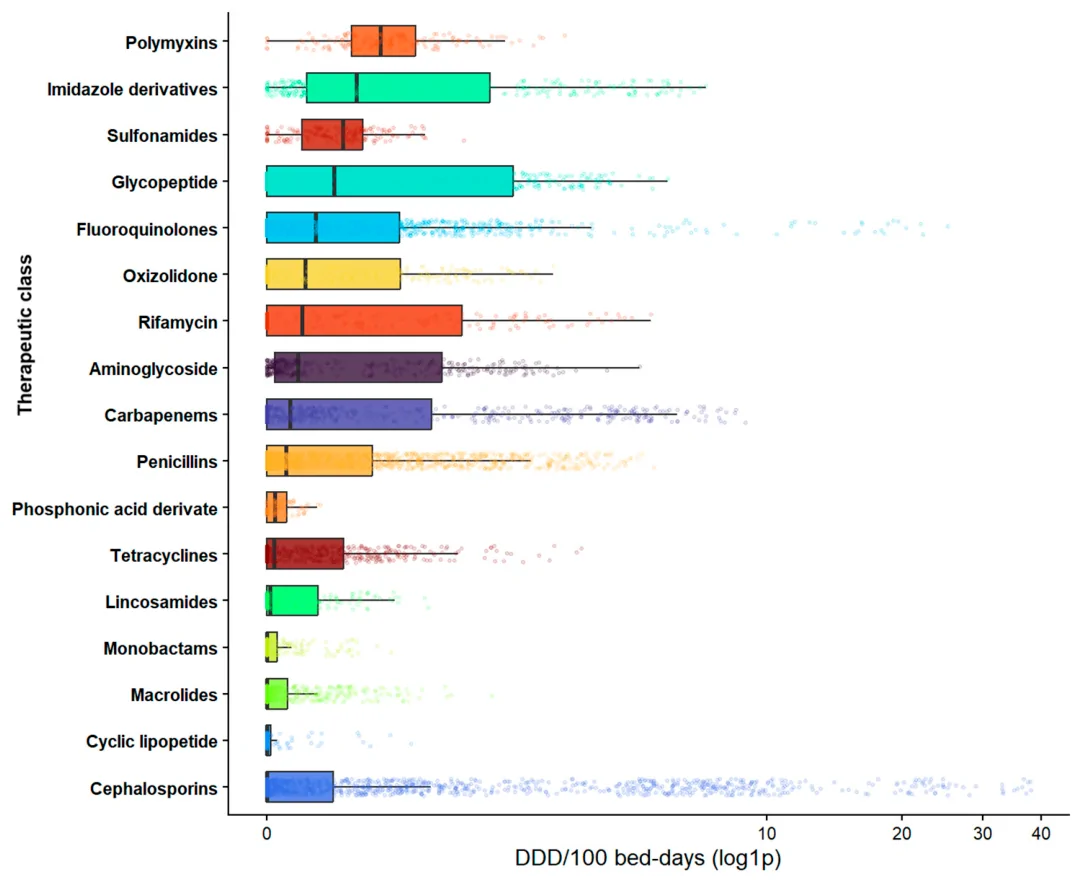
The distribution of overall antibiotic consumption by therapeutic group across the five study years.

The analysis of antibiotic consumption expressed by DDD/100 bed-days presented in Figure 11 showed statistically significant differences among the 17 therapeutic classes analyzed (Kruskal–Wallis test, *p* < 0.001). The corresponding omnibus effect size was ε^2^ = 0.119, indicating a modest overall difference among therapeutic classes. The highest consumption was for cephalosporins, fluoroquinolones, penicillins, and carbapenems. The median consumption values were 0.000 DDD/100 bed-days for cephalosporins, 0.266 DDD/100 bed-days for fluoroquinolones, 0.096 DDD/100 bed-days for penicillins, and 0.119 DDD/100 bed-days for carbapenems. The existence of significant differences between multiple pairs of therapeutic classes was confirmed by the post hoc analysis, using the Dunn test with Bonferroni correction of *p*-values, presented in the Supplementary Materials (Table S3). The most pronounced differences were observed between classes characterized by high distributions of the DDD/100 bed-day indicator and classes with predominantly low values. At the same time, the differences were no longer statistically significant after applying the correction for multiple comparisons for certain pairs of therapeutic groups.

Restricting the comparison to the four highest-consumption classes (cephalosporins, fluoroquinolones, penicillins, carbapenems) the omnibus effect size was epsilon-squared = 0.044, with pairwise rank-biserial correlations ranging from small (carbapenems versus penicillins, r = −0.01, 95% CI [−0.06, 0.04], not significant after correction) to small-to-moderate (cephalosporins versus fluoroquinolones, r = 0.27, 95% CI [0.23, 0.30]).

**Figure 12:**
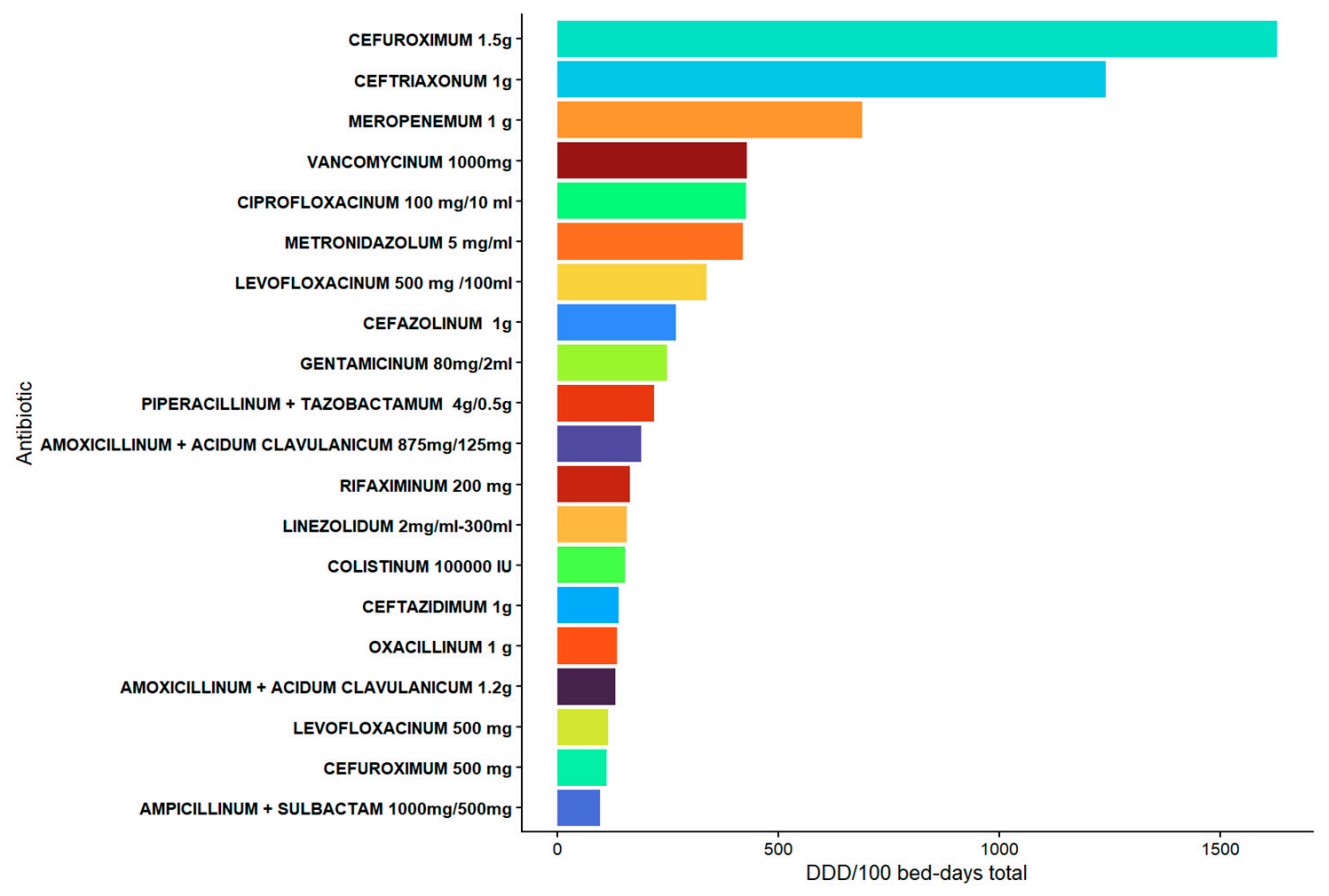
Top 20 antibiotics based on overall consumption expressed by DDD/100 bed-days.

The analysis of individual antibiotics showed a heterogeneous distribution of cumulative DDD/100 bed-days across the antibacterial agents included in the study (Figure 12). The predominance of cephalosporins among the highest-ranking individual antibiotics was consistent with the therapeutic class analysis. Among all evaluated antibiotics, cefuroxime 1.5 g accounted for the highest cumulative consumption (1627 DDD/100 bed-days), followed by ceftriaxone 1 g (1239 DDD/100 bed-days) and meropenem 1 g (690 DDD/100 bed-days). Other antibiotics with high cumulative consumption included ciprofloxacin (498 DDD/100 bed-days), metronidazole (491 DDD/100 bed-days), and vancomycin (429 DDD/100 bed-days).

**Figure 13:**
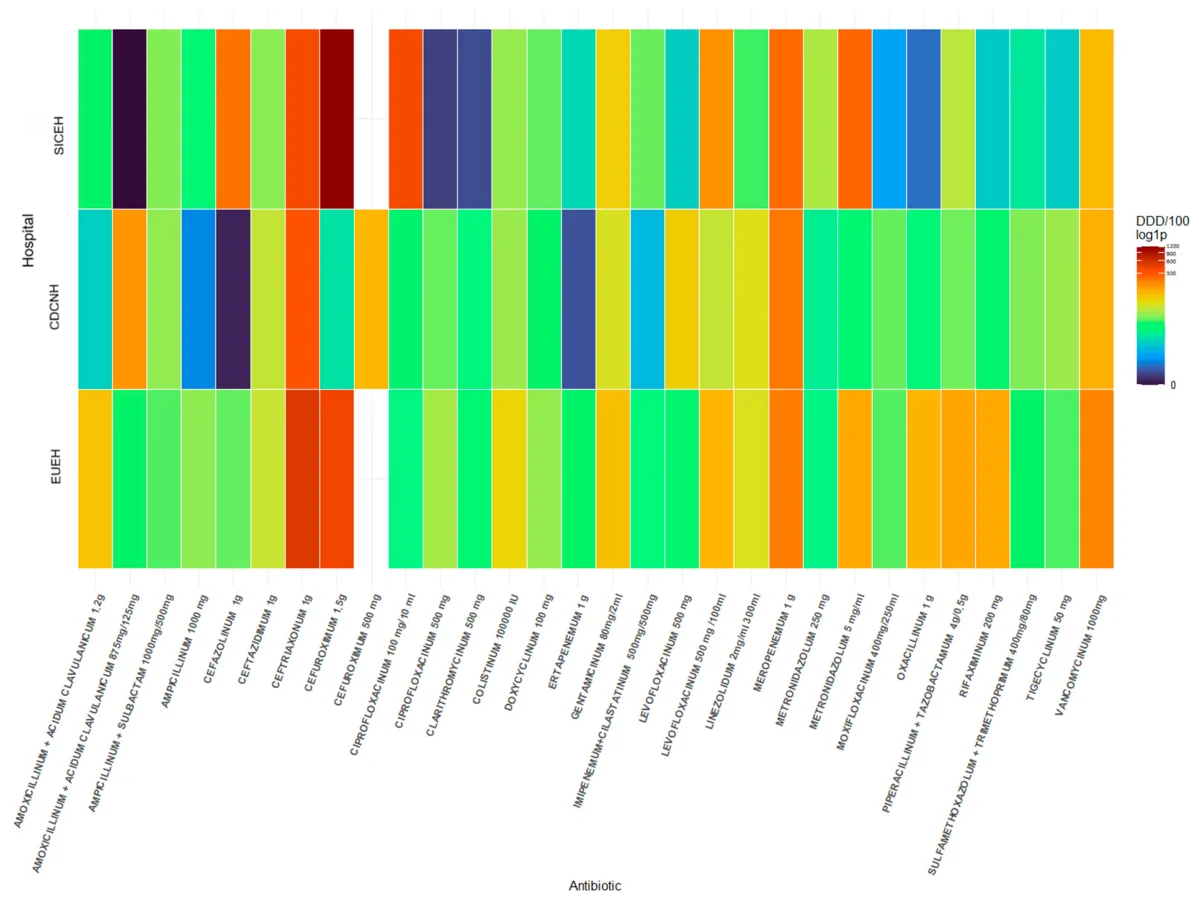
Comparative heatmap of top antibiotic consumption stratified by hospital. White space = antibiotic not utilized by the hospital; SICEH = Sf. Ioan Clinical Emergency Hospital; CDCNH = Dr. Carol Davila Clinical Nephrology Hospital; EUEH = Elias University Emergency Hospital; DDD = Daily Defined Dose.

As shown in Figure 13, antibiotic consumption patterns differed among the three hospitals in terms of both consumption levels and the predominant antibiotics. Elias Hospital exhibited relatively high values for several antibiotics: ceftriaxone, cefuroxime, and meropenem.

Sf. Ioan Hospital showed relatively high consumption values for cefazolin 1 g and ciprofloxacin 100 mg/mL compared with the other hospitals. Nephrology Hospital generally showed lower antibiotic consumption values, although relatively high consumption was observed for piperacillin/tazobactam.

Overall, the hospital-level results showed distinct antibiotic consumption profiles across the three facilities. EUEH had the highest median antibiotic consumption (0.234 DDD/100 bed-days), followed by CDCNH (0.024 DDD/100 bed-days), whereas SICEH had the lowest median value (0.000 DDD/100 bed-days). Watch antibiotics predominated in the AWaRe distribution across the participating hospitals, although the relative contribution of the AWaRe categories varied by hospital and study year. Cephalosporins represented the therapeutic class with the highest cumulative consumption in all three hospitals. At EUEH, the three leading classes were cephalosporins (1047.82 DDD/100 bed-days), penicillins (469.98 DDD/100 bed-days), and carbapenems (252.39 DDD/100 bed-days). The same three classes predominated at CDCNH, with cumulative consumption values of 525.41, 296.93, and 230.50 DDD/100 bed-days, respectively. At SICEH, cephalosporins showed the highest cumulative consumption (1884.81 DDD/100 bed-days), followed by fluoroquinolones (571.67 DDD/100 bed-days) and imidazole derivatives (307.73 DDD/100 bed-days).

The consumption distributions for the 12 selected antibiotics over the five-year period at each hospital are illustrated in the Supplementary Materials (Figures S5–S16). The analysis revealed differences in consumption at each hospital, with 33 out of 36 tests having statistically significant results (*p* < 0.05). Overall, significant differences in the consumption of the 12 selected antibiotics across study years were observed at each hospital, with the following exceptions: colistin (*p* = 0.62) and levofloxacin (*p* = 0.34) at Nephrology Hospital and imipenem/cilastatin at Elias Hospital (*p* = 0.64). The post hoc analysis is illustrated in the Supplementary Materials (Table S4).

Furthermore, the analysis conducted across the three facilities identified significant differences in 54 out of 60 tests, with *p* < 0.05. While consumption of almost all substances differed significantly between hospitals, notable exceptions included colistin in 2018 (*p* = 0.41), levofloxacin in 2023 (*p* = 0.91), linezolid in 2018 (*p* = 0.22) and 2023 (*p* = 0.14), and meropenem in 2023 (*p* = 0.46). The post hoc analysis is presented in the Supplementary Materials (Table S5). Detailed annual consumption values, expressed by DDD/100 bed-days, for each pharmacological class and selected antibiotics are provided in the Supplementary Materials (Table S6).

**Figure 14:**
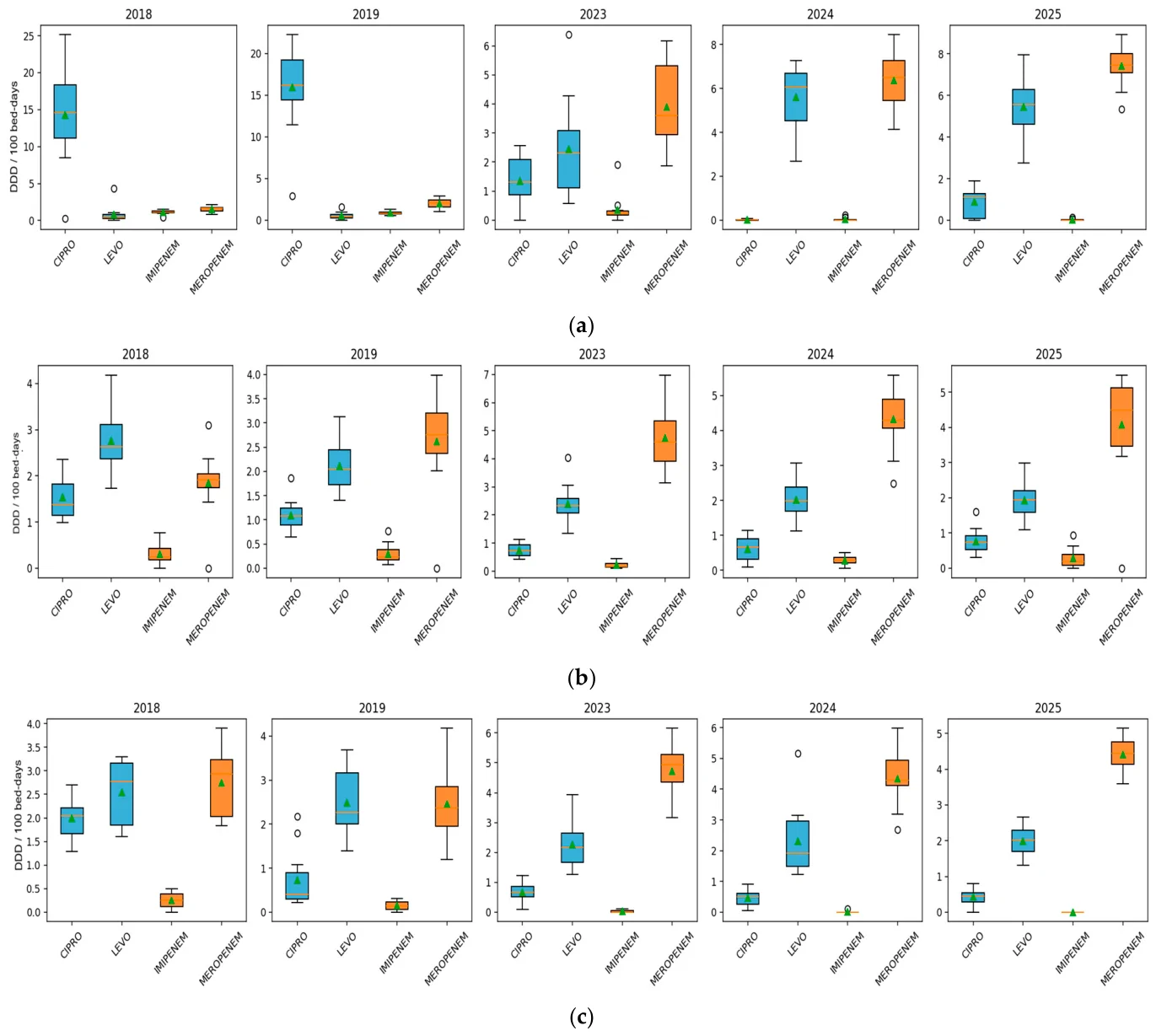
Antibiotic consumption analysis between ciprofloxacin, levofloxacin, meropenem, imipenem/cilastatin: (**a**) Sf. Ioan Hospital (**b**) Elias Hospital (**c**) Nephrology Hospital; CIPRO = Ciprofloxacin (500 mg, 100 mg/mL); LEVO = Levofloxacin (500 mg, 500 mg/mL); IMIPENEM = imipenem + cilastatin 500 mg/500 mg, MEROPENEM = meropenem 1 g. Boxplots show the median (orange line), mean (green triangle), interquartile range (box), and outliers (open circles).

Based on the observed differences in fluoroquinolone and carbapenem consumption across study years, an additional comparative analysis between these two antibiotic classes was performed. Comparisons between the representative fluoroquinolones (ciprofloxacin and levofloxacin) and carbapenems (imipenem + cilastatin and meropenem) demonstrated statistically significant differences (Mann–Whitney test) in antibiotic consumption across almost all hospital–year combinations (Figure 14). Of the 60 pairwise comparisons performed (3 hospitals × 5 years × 4 pairs), 55 (91.7%) were statistically significant after Holm correction (adjusted *p* < 0.05). Across the pooled fluoroquinolone–carbapenem comparisons, the rank-biserial correlation was r = 0.23 (95% CI [0.16, 0.30]), indicating a small-to-moderate effect. No statistically significant differences were observed for ciprofloxacin versus imipenem + cilastatin at Sf. Ioan Hospital in 2024, levofloxacin versus meropenem at Sf. Ioan Hospital in 2024 and Nephrology Hospital in 2018, or ciprofloxacin versus meropenem at Elias Hospital in 2018.

**Figure 15:**
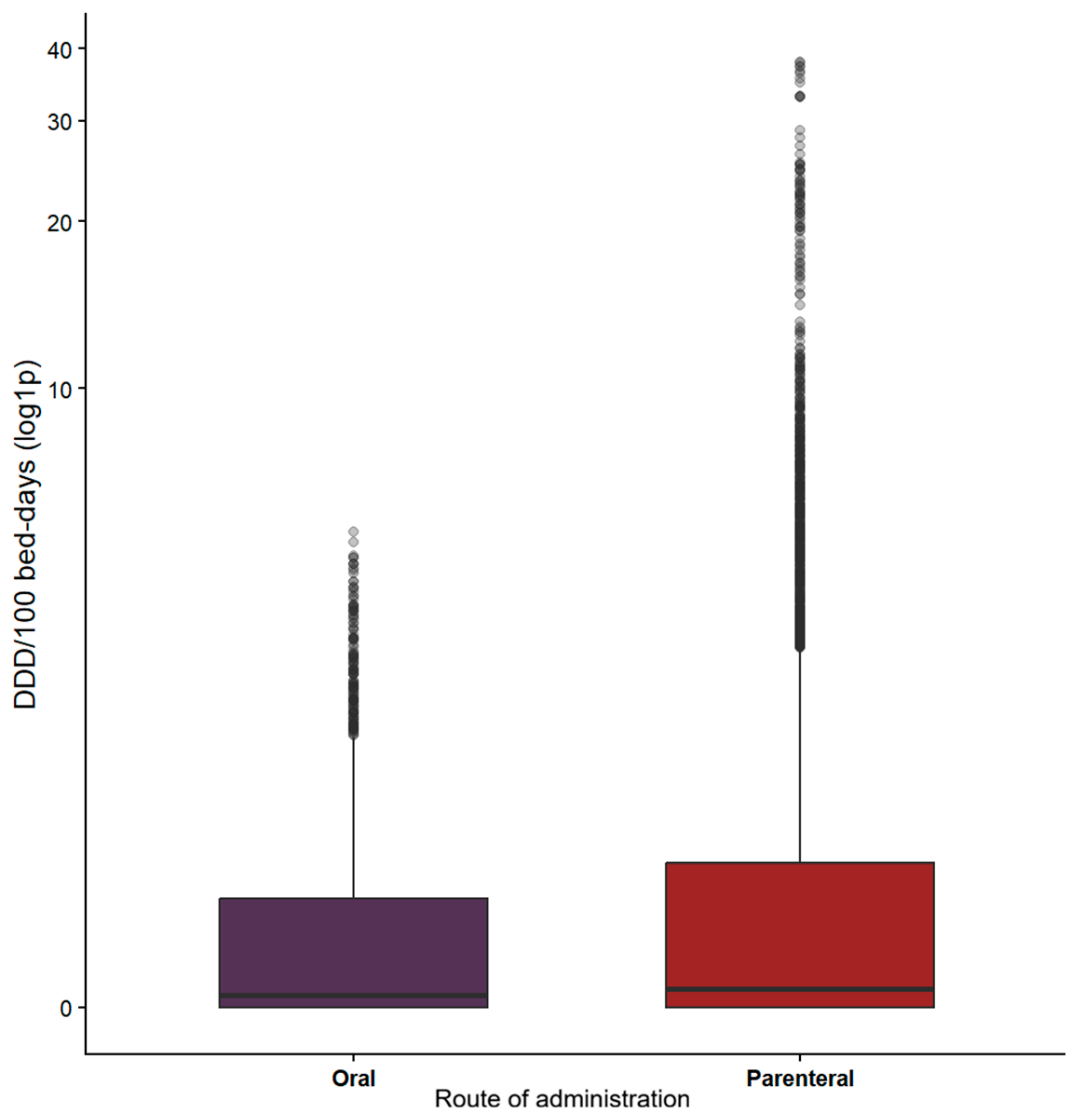
Distribution of overall antibiotic consumption by route of administration.

Median antibiotic consumption was 0.047 DDD/100 bed-days for orally administered antibiotics and 0.072 DDD/100 bed-days for parenterally administered antibiotics. The Mann–Whitney U test showed statistically significant differences between orally and parenterally administered antibiotics (*p* < 0.001). Despite the high statistical significance, the effect size was small (rank-biserial correlation r = 0.07, 95% CI [0.04, 0.09]), indicating that the route of administration accounts for only a modest share of the variability in DDD/100 bed-days. Parenteral formulations showed a wider distribution of DDD/100 bed-day values, with greater variability and higher extreme values compared to oral formulations (Figure 15).

## 3. Discussion

This five-year retrospective observational study provides an overview of antibiotic consumption patterns across three Romanian hospitals with distinct clinical profiles during 2018, 2019, 2023, 2024, and 2025.

Evaluating overall annual consumption showed differences in consumption patterns across the three hospitals and study years. Sf. Ioan Hospital showed relatively high annual antibiotic consumption throughout the study years. At Elias Hospital, annual consumption was higher in the later study years than in 2018 and 2019, while Nephrology Hospital maintained lower overall consumption levels, although higher values were observed in several later study years. These findings align with data from the ECDC annual report (2025), which indicates that hospital consumption of antibacterials for systemic use in Romania increased from 1.43 to 1.69 DDD/1000 inhabitants between 2020 and 2024 [21].

The monthly distribution of antibiotic consumption also differed across hospitals and study years. EUEH generally showed higher monthly consumption values and greater variability, whereas SICEH and CDCNH showed lower values. At CDCNH, monthly antibiotic consumption was lower in 2018 and 2019 than in several of the later study years, with greater variability observed during the period of 2023–2025. SICEH showed comparatively lower monthly consumption values, although variation was observed across individual months and study years. Medicine shortages may also influence patterns of antibiotic availability and use, and recent evidence has highlighted structural patterns of systemic antibacterial shortages across different countries [22]. These differences should be interpreted cautiously, as the available aggregated consumption data do not allow for the identification of the clinical, microbiological, or organizational factors underlying the observed patterns.

An analysis of inpatient antibacterial consumption in Belgium revealed an increase between 2017 and 2022 for consumption measured in DDD/1000 admissions and DDD/1000 patient-days [23]. The COVID-19 pandemic also affected the implementation of antimicrobial stewardship programs, highlighting the importance of continued surveillance of antibiotic consumption and prescribing practices. These findings further emphasize the value of continued surveillance of antibiotic consumption and prescribing practices, particularly following periods of major disruption to healthcare systems.

Correlation analysis between hospital bed-days and standardized antibiotic consumption showed significant positive associations at Sf. Ioan Hospital and Nephrology Hospital over the five-year study period, whereas no significant association was observed at Elias Hospital. Evaluating antibiotic consumption using the WHO AWaRe classification revealed a reliance on broad-spectrum agents across all three participating hospitals. Across the study years, Watch antibiotics dominated overall consumption, accounting for 62% to 82% of annual rates. Our findings identified differences in the utilization of Reserve antibiotics. At the Nephrology Hospital, usage nearly tripled, rising from 4.7% in 2018 to a peak of 13% in 2024. Similarly at Elias Hospital, Reserve antibiotics accounted for 4.2% in 2018 and 8.5% in 2024. The rates for Access group remained low across all facilities (16–31%), below the WHO stewardship target of >60%. Further research regarding clinical and microbiological data are required to determine the factors associated with the use of broad-spectrum therapies. Notably, antibiotics from the “Not Recommended” group were not recorded throughout the five-year period at Sf. Ioan Hospital. At Elias Hospital and the Nephrology Hospital, this category was represented by the combination of cefoperazone and sulbactam, which was present all five years. The ECDC Annual Epidemiological Report for 2024 indicated that the consumption of Reserve antibiotics in Romanian hospitals ranged from 2.5% in 2020 to 3.4% in 2024, remaining below the mean EU/EEA values (4.0% to 5.4%) [21]. A study of antimicrobial consumption across 721 hospitals in 73 countries (2015, 2017, 2018) revealed that in Eastern Europe, the Access group accounted for 30.29% of antibiotics, the most commonly prescribed agents being Watch representatives: ceftriaxone (24.8%) and ciprofloxacin (9.7%) [24].

Our findings revealed inter-hospital variability in antibiotic consumption rates across the included facilities. Notably, high utilization of broad-spectrum agents, predominantly from the Watch category, including cephalosporins (ceftriaxone, cefuroxime), carbapenems (meropenem), and parenteral fluoroquinolones (levofloxacin) was observed at Sf. Ioan and Elias Hospitals. High consumption of these agents was observed alongside frequent utilization of Access penicillins (amoxicillin/clavulanic acid) and metronidazole.

The high consumption of Watch antibiotics is consistent with the data reported by the ECDC. Hospital sectors across Central and Eastern Europe show higher proportions of Watch antibiotic prescribing compared to Western European consumption [21]. However, a study conducted in a Romanian hospital reported an increase in Access antibiotics in 2023 (21%) and 2024 (41%) in comparison with 2020–2022 (below 10%) [25].

Furthermore, Elias Hospital recorded higher consumption levels of antistaphylococcal antibiotics (piperacillin/tazobactam and oxacillin) and gastro-intestinal antibacterials (rifaximin) than the other two facilities.

Meropenem, vancomycin, and ceftriaxone, all classified as Watch antibiotics, showed high consumption rates across all three hospitals. A study conducted in a Romanian hospital revealed high prescribing rates for meropenem and vancomycin in 2022, followed by a decline in 2023 and 2024 [26].

Fluoroquinolone consumption was lower in the later study years, predominantly involving ciprofloxacin at Sf. Ioan and Elias Hospitals and levofloxacin at the Nephrology and Elias hospitals. This reduction in consumption is similar to findings reported in studies on European prescribing practices. The European Medicines Agency issued safety warnings in 2018–2019, advising the restriction of systemic fluoroquinolones due to concerns regarding long-lasting and disabling adverse reactions [27]. These regulatory actions have been reflected in a reduction in fluoroquinolone prescribing across Europe. A study about a network utilized for quarterly antibiotic-use surveillance showed for the period September 2017–2019 a decline in fluoroquinolone use, followed by an increase in 2020 [28]. Furthermore, a study conducted in a multidisciplinary hospital in Romania revealed an overall reduction in ciprofloxacin in the period 2020–2024, alongside a decline in levofloxacin in 2024 compared with the increased consumption during the period of 2020–2023 [25].

Conversely, higher carbapenem consumption was observed in the later study years. Carbapenems often remain a treatment option for infections caused by multidrug-resistant Gram-negative organisms, particularly extended-spectrum β-lactamase (ESBL)-producing *Enterobacterales* [29]. At the individual hospital level, meropenem (Watch group) consumption was higher in the later study years across all three hospitals.

The consumption of ceftazidime/avibactam showed pronounced differences across study years, with almost no utilization in 2018–2019 across all three hospitals. A similar increase was reported in another Romanian hospital, from 0.02 DDD/100 bed-days in 2022 to 0.26 DDD/100 bed-day in 2024 [25]. Ceftriaxone consumption remained relatively constant across included years at Nephrology Hospital, with higher values at Elias Hospital. However, marked variation in ceftriaxone consumption was observed across study years at Sf. Ioan Hospital. Cefuroxime also showed substantial variation across study years at this hospital.

Colistin (polymyxins, Reserve group) consumption increased at Elias Hospital from 2018 to 2023 (peak), followed by a decline, while consumption remained low and relatively stable at the other two hospitals. Different consumption patterns were observed for gentamicin (Access group): a progressive decline at Sf. Ioan Hospital and an increased consumption at the Nephrology Hospital until 2023 (peak), followed by a decrease. Meanwhile, Elias Hospital displayed a continuous reduction.

Linezolid (oxazolidinones, Reserve group) utilization also showed distinct temporal patterns across study years in the included hospitals: an increase from 2018 to 2025 at Nephrology Hospital, with low values at Sf. Ioan.

Antimicrobial resistance and healthcare-associated infections represent a significant public health challenge globally. In recent years, international initiatives have been undertaken to optimize antibiotic prescribing and lower resistance rates, ranging from the establishment of monitoring programs (WHO Global Antimicrobial Resistance and Use Surveillance System strategies), preparedness frameworks that integrate the One Health approach, to the monitoring of supply chains for critical antibiotics [30,31,32]. According to a report published by the Organization for Economic Cooperation and Development, the implementation of antimicrobial stewardship programs and infection control measures could prevent 4.8 million deaths by 2050 [33].

In alignment with international directives, Romania has implemented public health policies to promote rational prescribing practices and mitigate AMR risks. The National Health Strategy for 2023–2030 prioritizes the prevention of healthcare-associated infections, resistance monitoring and the establishment of multidisciplinary antimicrobial stewardship teams in hospitals [34]. Law no. 3 from 2021 establishes guidelines for the rational use of antibiotics and the prevention of antimicrobial resistance in hospitals [35]. Moreover, hospital pharmacists are responsible for performing monthly monitoring and reporting of inpatient antibiotic consumption, expressed in DDD/100 bed-days [36]. Another initiative to optimize antibiotic therapy involved the introduction of an antibiotic restriction formulary. In 2023, Sf. Ioan Hospital implemented a restricted antibiotic dispensing policy for a predefined list of selected broad-spectrum antibiotics approved by the Hospital Medical Council. Dispensing these antibiotics required documented clinical justification, microbiological investigations when available, and approval in accordance with institutional procedures.

This intervention is similar to one reported in another study conducted in Romania (2022–2024). The study showed that the intervention resulted in a reduction in the consumption of Reserve antibiotics, although overall consumption remained constant [26]. The implementation of administrative interventions can reduce antibiotic consumption, as reported in a study performed in a tertiary general hospital [37].

Regarding the route of administration, parenteral formulations had the highest consumption rates, consistent with the results reported by a study conducted in a Romanian hospital during the period of 2017–2021 [20].

Limitations: The study has some limitations. The analysis included only three hospitals from a single geographical region, making it difficult to extend the results on national level. Antibiotic consumption data were unavailable for 2020–2022 because of pandemic-related changes in hospital organization, resulting in a discontinuity in the observation period. Consequently, the study does not permit continuous longitudinal assessment across the entire 2018–2025 period or an evaluation of the impact of the COVID-19 pandemic on antibiotic consumption. Antibiotic consumption was evaluated using data expressed as DDD/100 bed-days. Consequently, information about the duration of therapy, appropriateness of treatment, microbiological analyses, and clinical outcomes was not available. Indicators were based on DDD quantify antibiotic consumption without accounting for individual dose adjustments or specific clinical circumstances.

Furthermore, consumption data were collected from hospital pharmacy electronic records, reflecting antibiotics dispensed by the hospital pharmacies rather than actual administration to individual patients. Consequently, discrepancies between dispensed and administered quantities cannot be excluded.

Annual correlation analyses were calculated for each year separately based on 12 repeated monthly observations per hospital. These monthly values are repeated measurements of the same underlying process in the same hospital rather than independent measurements. Consequently, temporal autocorrelation between consecutive months was not accounted for and may have influenced the estimated statistical significance, so annual correlation results should be interpreted with caution. For this reason, we also reported the overall correlation analysis across the five-year period (*n* = 60 monthly observations per hospital), together with the comparison across calendar months (Figure S1). Both offer a more stable picture of the association, although neither solves the limitation completely. Time-series models that treat autocorrelation and seasonality explicitly would be the appropriate approach, and we intend to apply them in further work.

Another limitation concerns the number of tests performed. Several hundred comparisons were carried out in total (across the three hospitals, the five years, four AWaRe categories, the therapeutic classes, and the 12 individual antibiotics). The corrections used (Bonferroni for Dunn’s post hoc tests, Holm for the pairwise Mann–Whitney comparisons) were applied inside each family of related comparisons, not once over the whole dataset. This is the usual practice in antibiotic consumption studies, but it does not remove the cumulative Type I error at the level of the manuscript. We have treated isolated results and those with *p* close to 0.05 as hypothesis-generating. They require confirmation on larger datasets.

Despite these limitations, the study provides a comprehensive overview of antibiotic consumption patterns in three Romanian tertiary-care hospitals and establishes a valuable baseline for future antimicrobial stewardship initiatives and national monitoring programs. The integration of quantitative consumption data with the WHO AWaRe framework may also facilitate future comparisons with other European hospitals and contribute to monitoring antibiotic consumption over time.

## 4. Materials and Methods

### 4.1. Study Design and Setting

A retrospective, multicenter study was conducted to evaluate patterns of systemic antibiotic consumption in three hospitals in Bucharest, Romania. The study included five years: 2018, 2019, 2023, 2024, and 2025. Antibiotic consumption data were unavailable for 2020–2022 because of pandemic-related changes in hospital organization. The COVID-19 pandemic affected hospital antimicrobial consumption and the implementation of antimicrobial stewardship and infection prevention and control programs [16].

The participating hospitals were Sf. Ioan Clinical Emergency Hospital, Elias University Emergency Hospital, and Dr. Carol Davila Clinical Nephrology Hospital, each with a distinct clinical profile. Sf. Ioan Clinical Emergency Hospital (SICEH) is a large multidisciplinary emergency hospital providing medical, surgical, obstetric, and intensive care services, with 631 beds, including 41 intensive care beds and more than 25,000 hospital admissions annually. Elias University Emergency Hospital (EUEH) is a multidisciplinary university emergency hospital offering a wide range of medical and surgical specialties, with 913 inpatient beds, including 30 intensive care beds and approximately 25,000–30,000 inpatient admissions/discharges annually. Dr. Carol Davila Clinical Nephrology Hospital (CDCNH) is a specialized nephrology hospital providing nephrology, dialysis, general surgical, and intensive care services, with 208 beds, including 15 intensive care beds and approximately 10,000–12,000 admissions/discharges annually. Its Hemodialysis Center is equipped with 11 dialysis machines, and dialysis activity was included in the study. The inclusion of hospitals with diverse clinical profiles enabled comparisons of antibiotic consumption patterns across different healthcare settings.

None of the participating hospitals had a formally established multidisciplinary antimicrobial stewardship team during the study period; however, institutional procedures for restricting selected antibiotics were in place. At Sf. Ioan Hospital, a restricted dispensing policy for selected broad-spectrum antibiotics was implemented in 2023. At Elias Hospital, antibiotics classified in the WHO AWaRe Reserve group were restricted and could be dispensed only with the approval of an infectious disease physician. At Nephrology Hospital, a predefined list of restricted antibiotics was used for last-resort treatment of infections caused by multidrug-resistant organisms, and dispensing required approval from an infectious disease physician and the medical director.

### 4.2. Data Collection and Antibiotic Consumption

Antibiotic consumption data were extracted from the hospital pharmacies’ electronic system reports from all wards. The pharmacy records reflected antibiotics dispensed for individual patients but did not provide confirmation of actual administration to patients. A database was created by harmonizing the pharmacy datasets from all participating facilities, including for each antibiotic the monthly number of therapeutic units.

Each antibacterial agent was classified according to the World Health Organization (WHO) Anatomical Therapeutic Chemical (ATC) system. The analysis included pharmaceutical products for systemic use based on active substances from the group J01 (antibacterials for systemic use) [38].

Antibiotic use was quantified using the WHO Anatomical Therapeutic Chemical/Defined Daily Dose (ATC/DDD) methodology, the internationally accepted standard for antimicrobial utilization surveillance [39]. The Defined Daily Dose (DDD) represents the assumed average maintenance dose per day for a medicine used for its principal indication. The DDD was calculated for each antibiotic by dividing the total amount of antibiotic used (g) by the individual DDD value defined by WHO for that antibiotic, using the WHO ATC/DDD Index [38]. Antibiotic consumption was then standardized by calculating DDD per 100 bed-days using monthly hospital bed-days as the denominator. A bed-day was defined as a day of continuous inpatient hospitalization involving an overnight stay. Day-case admissions were not included in the bed-day denominator. This indicator enabled comparisons of antibiotic utilization between hospitals with different patient volumes and levels of clinical activity. Monthly hospital bed-days were collected separately for each hospital [40].

All antibiotics included in the study were assigned to their respective WHO AWaRe categories (Access, Watch, Reserve, and Not Recommended) [41].

The study was approved by the Ethics Committees from Sf. Ioan Hospital and Dr. Carol Davila Clinical Nephrology Hospital and by the hospital manager at Elias Hospital.

Only anonymized pharmacy consumption data were analyzed. No patient-identifiable information was collected, processed or reported.

### 4.3. Statistical Analysis

The statistical analysis was performed using the open-source R software (version 4.6.0) [42]. Antibiotic consumption was expressed as DDD/100 bed-days.

Antibiotic consumption across hospitals, study years, therapeutic classes, WHO AWaRe categories, and individual antibacterial agents was compared using the Kruskal–Wallis non-parametric test. It was followed by post hoc pairwise comparisons to identify groups that differed (Dunn’s test with Bonferroni correction and Mann–Whitney U test with Holm correction). Corrections for multiple testing were applied within each family of related comparisons rather than globally across all analyses. Additionally, the Mann–Whitney U test was used to compare consumption distribution according to the route of administration. Statistical analyses were performed on raw DDD/100 bed-day values. Logarithmic transformation (log_1p_) was applied for graphical visualization to account for extreme outliers or low consumption values (<1 DDD/100 bed-days).

Although corrections for multiple testing were applied within each family of related comparisons, no global adjustment was performed across all statistical analyses. An increased cumulative risk of Type I error cannot be excluded due to the large number of comparisons performed. Therefore, isolated or borderline significant findings should be interpreted with caution. Furthermore, statistical analyses should be considered primarily exploratory.

For each hospital, temporal patterns in antibiotic consumption were described using the monthly median as the primary summary statistic, which is a robust estimator for datasets containing outliers or periods of reduced consumption. Monthly data were aggregated to calculate the overall median DDD/100 bed-days for each facility.

Associations between monthly hospital bed-days and antibiotic consumption density were evaluated separately for each hospital. Annual correlation analyses were based on 12 monthly observations per hospital (*n* = 12), whereas the overall analysis across the five study years included 60 monthly observations per hospital (*n* = 60). Bivariate correlation analyses were performed using Pearson’s correlation coefficient (r) for linear relationships and Spearman’s rank correlation coefficient (rho) to account for non-linear distributions. As the annual correlation analyses relied on repeated monthly measurements from the same hospital rather than independent observations, potential autocorrelation between consecutive months was considered when interpreting these results and mentioned as a limitation.

Twelve antibiotics were selected for individual analysis. Selection was based on their frequency and clinical relevance across the participating hospitals while also ensuring representation of major therapeutic classes and the WHO AWaRe categories: cefuroxime, ceftriaxone, meropenem, imipenem/cilastatin, ciprofloxacin, levofloxacin, piperacillin/tazobactam, vancomycin (Watch group), linezolid, ceftazidime/avibactam and colistin (Reserve group), and gentamicin (Access group). The Kruskal–Wallis test was used on 12 monthly values per group, followed by a post hoc Mann–Whitney test with Holm correction where significant differences were identified. We analyzed antibiotic consumption by hospital over the five-year period and also conducted comparative assessments between hospitals for each year. No a priori power or sample-size calculation was performed because the analysis included the complete set of available pharmacy records from the participating hospitals for the predefined study years rather than a sample drawn from a larger dataset. The statistical analyses were therefore considered exploratory and descriptive rather than confirmatory.

We have calculated effect sizes with 95% confidence intervals for the primary comparisons reported in the text, using the underlying dataset: epsilon-squared for the Kruskal–Wallis omnibus tests, rank-biserial correlations (with bootstrap 95% CI) for the pairwise Mann–Whitney/Dunn comparisons, and Fisher-z 95% confidence intervals for all reported Pearson correlation coefficients. Statistical significance was set at *p* < 0.05.

## 5. Conclusions

The findings demonstrated substantial differences in antibiotic consumption patterns, expressed as DDD/100 bed-days, across hospitals, therapeutic classes, and study years. Statistically significant differences were identified among the WHO AWaRe categories, with Access antibiotics accounting for less than 32% of annual consumption, below the WHO target of 60%.

Continuous monitoring can support antimicrobial stewardship programs by identifying antibiotic consumption patterns, evaluating changes over time, and informing authorities to promote the appropriate use of antibiotics. Antibiotic consumption monitoring should remain a priority for hospitals, as optimizing antimicrobial use represents a fundamental component of national efforts to contain antimicrobial resistance.

## Figures and Tables

**Table 1:** Antibiotic consumption based on AWaRe classification by year and hospital.

DDD/100 Bed-Days (% Total Annual Consumption)						
Hospital	AWaRe Group	2018	2019	2023	2024	2025
SICEH	Access	112.3 (16.0%)	117.1 (16.0%)	198.8 (31.8%)	126.3 (18.3%)	201.7 (26.4%)
	Watch	578.2 (82.4%)	600.7 (82.3%)	401.7 (64.2%)	551.2 (79.6%)	545.5 (71.4%)
	Reserve	11.1 (1.6%)	12.5 (1.7%)	24.9 (4.0%)	14.6 (2.1%)	16.9 (2.2%)
	Not Recommended	0.0 (0.0%)	0.0 (0.0%)	0.0 (0.0%)	0.0 (0.0%)	0.0 (0.0%)
	Total	701.6	730.3	625.4	692.1	764.1
EUEH	Access	81.6 (18.7%)	118.5 (25.5%)	149.8 (22.8%)	165.4 (24.7%)	184.4 (27.9%)
	Watch	335.3 (77.0%)	312.2 (67.3%)	452.9 (68.9%)	446.4 (66.7%)	430.3 (65.0%)
	Reserve	18.5 (4.2%)	33.3 (7.2%)	54.3 (8.3%)	57.2 (8.5%)	47.4 (7.2%)
	Not Recommended	0.2 (0.0%)	0.1 (0.0%)	0.0 (0.0%)	0.0 (0.0%)	0.0 (0.0%)
	Total	435.6	464.1	657	669	662.1
CDCNH	Access	78.6 (25.0%)	63.1 (22.6%)	88.3 (23.2%)	100.9 (24.5%)	94.1 (24.9%)
	Watch	220.8 (70.3%)	193.7 (69.3%)	263.3 (69.2%)	258.2 (62.6%)	236.3 (62.4%)
	Reserve	14.7 (4.7%)	22.8 (8.1%)	28.8 (7.6%)	53.4 (13.0%)	48.1 (12.7%)
	Not Recommended	0.0 (0.0%)	0.0 (0.0%)	0.1 (0.0%)	0.0 (0.0%)	0.0 (0.0%)
	Total	314.1	279.6	380.5	412.5	378.5

EUEH = Elias University Emergency Hospital; CDCNH = Dr. Carol Davila Clinical Nephrology Hospital; SICEH = Sf. Ioan Clinical Emergency Hospital; DDD = Daily Defined Dose.

## Data Availability

Data are contained within the article and Supplementary Materials.

## Supplementary Materials

The following supporting information can be downloaded at https://www.mdpi.com/article/10.3390/antibiotics15090901/s1, Figure S1. Seasonal variation in antibiotic use during 2018–2019 and 2023–2025 (1—January, 12—December) periods; Table S1. Pairwise post hoc comparison of total antibiotic consumption across AWaRe groups; Table S2. Pairwise post hoc comparison of AWaRe antibiotic consumption across study years within each hospital; Figure S2. Distribution of antibiotic consumption by AWaRe categories by year at Elias Hospital; Figure S3. Distribution of antibiotic consumption by AWaRe categories by year at Nephrology Hospital; Figure S4. Distribution of antibiotic consumption by AWaRe categories by year at Sf. Ioan Hospital; Table S3. Pairwise post hoc comparison of overall antibiotic consumption across pharmacological classes; Figure S5. Distribution of Ceftazidime/avibactam consumption across hospitals and study years; Figure S6. Distribution of Ceftriaxone consumption across hospitals and study years; Figure S7. Distribution of Cefuroxime consumption across hospitals and study years; Figure S8. Distribution of Ciprofloxacin consumption across hospitals and study years; Figure S9. Distribution of Levofloxacin consumption across hospitals and study years; Figure S10. Distribution of Colistin consumption across hospitals and study years; Figure S11. Distribution of Gentamicin consumption across hospitals and study years; Figure S12. Distribution of Imipenem/cilastatin consumption across hospitals and study years; Figure S13. Distribution of Linezolid consumption across hospitals and study years; Figure S14. Distribution of Meropenem consumption across hospitals and study years; Figure S15. Distribution of Piperacillin/tazobactam consumption across hospitals and study years; Figure S16. Distribution of Vancomycin consumption across hospitals and study years; Table S4. Pairwise post hoc comparison of 12 selected antibiotic consumption across study years within each hospital; Table S5. Pairwise post hoc comparison of 12 selected antibiotic consumption across hospitals for each study year. Table S6. Annual antibiotic consumption by pharmacological class and selected substances across hospitals.

## Abbreviations

The following abbreviations are used in this manuscript:

AMR	Antimicrobial resistance
ATC	Anatomical Therapeutic Chemical
ASP	Antimicrobial stewardship program
AWaRe	Access, Watch, and Reserve
CDCNH	Dr. Carol Davila Clinical Nephrology Hospital
DDD	Defined Daily Dose
ECDC	European Centre for Disease Prevention and Control
ESBL	Extended-spectrum β-lactamase
EU/EEA	European Union/European Economic Area
EUEH	Elias University Emergency Hospital
SICEH	Sf. Ioan Clinical Emergency Hospital
MDR	Multidrug-resistant
WHO	World Health Organization
