# Supplementary material for: Antibiotic Consumption Patterns in Three Romanian Hospitals: A Five-Year Retrospective Study
Source: Antibiotics (Basel). 2026 Sep 13;15(9):901. doi: 10.3390/antibiotics15090901 (PMC13603202; doi:10.3390/antibiotics15090901)
Supplement: Supplementary file 1 [file antibiotics-15-00901-s001.zip › antibiotics-4512455-supplementary.pdf]

Supplementary Materials

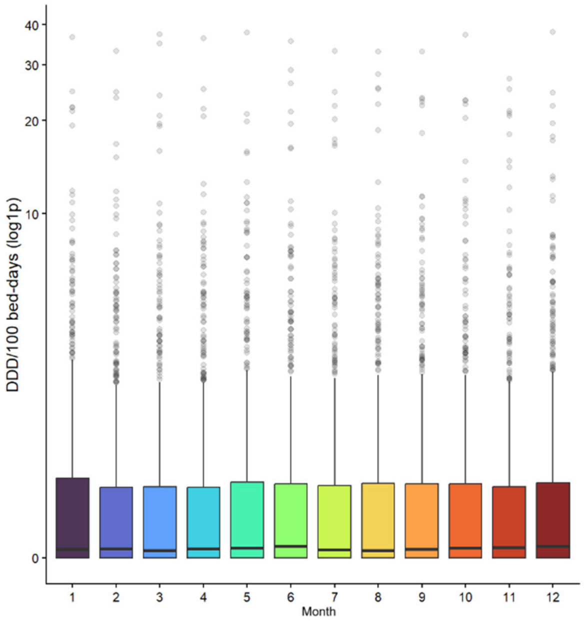

Figure S1. Seasonal variation in antibiotic use during 2018-2019 and 2023-2025 (1- January, 12- December) periods.

Table S1. Pairwise post hoc comparison of total antibiotic consumption across AWaRe groups.

Group 1	Group 2	n1	n2	Statistic Test[a]	p-value[a]	Adjusted p-value (p.adj)[b]	Significance (p.adj.signif)
Access	Not recommended	2925	180	-15.12	< 0.0001	< 0.0001	****
Access	Reserve	2925	2194	-21.36	< 0.0001	< 0.0001	****
Access	Watch	2925	4214	-2.80	0.0051	0.0305	*
Not recommended	Reserve	180	2194	7.19	< 0.0001	< 0.0001	****
Not recommended	Watch	180	4214	14.37	< 0.0001	< 0.0001	****
Reserve	Watch	2194	4214	20.36	< 0.0001	< 0.0001	****

n1, n2= sample sizes for analysed groups; [a] post hoc pairwise comparisons were performed using the Dunn test; [b] Adjusted p-values for multiple testing using Bonferroni correction method; antibiotic consumption was expressed as DDD / 100 bed-days; significance level: * $p < 0.05$, **** $p < 0.0001$.

Table S2. Pairwise post hoc comparison of AWaRe antibiotic consumption across study years within each hospital.

AWaRe Group	Hospital	Year 1	Year 2	p-value[a]	Adjusted p-value (p_holm)[b]	Significant
Access	SICEH	2018	2023	0.0004	0.0038	Yes
Access	SICEH	2018	2025	0.0011	0.0088	Yes
Access	SICEH	2019	2023	0.0007	0.0066	Yes
Access	SICEH	2019	2025	0.0017	0.0116	Yes
Access	CDCNH	2018	2019	0.0043	0.0299	Yes
Access	CDCNH	2018	2024	0.0043	0.0299	Yes
Access	CDCNH	2019	2023	0.0003	0.0025	Yes
Access	CDCNH	2019	2024	< 0.0001	0.0005	Yes
Access	CDCNH	2019	2025	< 0.0001	0.0004	Yes
Access	EUEH	2018	2019	0.0001	0.0007	Yes
Access	EUEH	2018	2023	< 0.0001	0.0004	Yes
Access	EUEH	2018	2024	< 0.0001	0.0004	Yes
Access	EUEH	2018	2025	< 0.0001	0.0004	Yes
Access	EUEH	2019	2023	0.0007	0.0029	Yes
Access	EUEH	2019	2024	0.0002	0.0010	Yes
Access	EUEH	2019	2025	< 0.0001	0.0004	Yes
Access	EUEH	2023	2024	0.0102	0.0258	Yes
Access	EUEH	2023	2025	0.0086	0.0258	Yes
Watch	SICEH	2018	2023	0.0016	0.0149	Yes
Watch	SICEH	2019	2023	0.0007	0.0073	Yes
Watch	CDCNH	2018	2019	0.0061	0.0305	Yes
Watch	CDCNH	2018	2023	0.0011	0.0077	Yes
Watch	CDCNH	2018	2024	0.0042	0.0256	Yes
Watch	CDCNH	2019	2023	< 0.0001	0.0004	Yes
Watch	CDCNH	2019	2024	< 0.0001	0.0004	Yes
Watch	CDCNH	2019	2025	0.0007	0.0059	Yes
Watch	EUEH	2018	2023	< 0.0001	0.0005	Yes
Watch	EUEH	2018	2024	0.0001	0.0006	Yes
Watch	EUEH	2018	2025	< 0.0001	0.0005	Yes
Watch	EUEH	2019	2023	< 0.0001	0.0004	Yes
Watch	EUEH	2019	2024	< 0.0001	0.0004	Yes
Watch	EUEH	2019	2025	< 0.0001	0.0004	Yes
Reserve	CDCNH	2018	2023	0.0005	0.0019	Yes
Reserve	CDCNH	2018	2024	< 0.0001	0.0004	Yes
Reserve	CDCNH	2018	2025	< 0.0001	0.0004	Yes
Reserve	CDCNH	2019	2024	< 0.0001	0.0004	Yes
Reserve	CDCNH	2019	2025	< 0.0001	0.0004	Yes
Reserve	CDCNH	2023	2024	0.0003	0.0015	Yes
Reserve	CDCNH	2023	2025	0.0003	0.0015	Yes
Reserve	EUEH	2018	2019	0.0004	0.0023	Yes
Reserve	EUEH	2018	2023	< 0.0001	0.0004	Yes

Reserve	EUEH	2018	2024	< 0.0001	0.0004	Yes
Reserve	EUEH	2018	2025	< 0.0001	0.0004	Yes
Reserve	EUEH	2019	2023	0.0005	0.0024	Yes
Reserve	EUEH	2019	2024	0.0002	0.0017	Yes
Reserve	EUEH	2019	2025	0.0020	0.0080	Yes

SICEH= Sf. Ioan Clinical Emergency Hospital; CDCNH= Dr. Carol Davila Clinical Nephrology Hospital; EUEH= Elias University Emergency Hospital; [a] post hoc pairwise comparisons were performed using the Mann–Whitney U test; [b] Adjusted p-values for multiple testing with the Holm correction method; antibiotic consumption was expressed as DDD / 100 bed-days; *Only statistically significant pairwise comparisons were presented in the table (adjusted p-value <0.05); non-significant comparisons were omitted to optimize space.

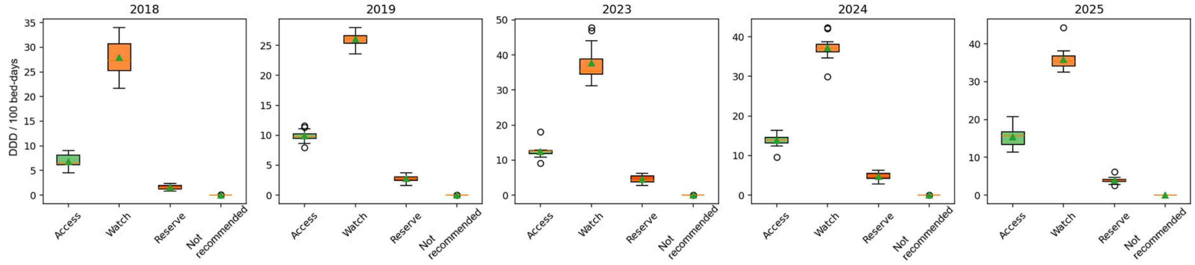

Figure S2. The distribution of antibiotic consumption by AWaRe categories by year at Elias Hospital. The boxplots show the median (orange line), mean (green triangle), interquartile range (box), and outliers (open circles).

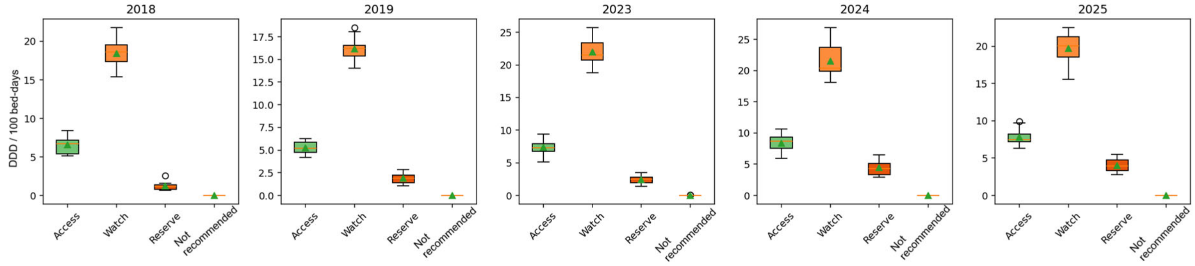

Figure S3. The distribution of antibiotic consumption by AWaRe categories by year at Nephrology Hospital. The boxplots show the median (orange line), mean (green triangle), interquartile range (box), and outliers (open circles).

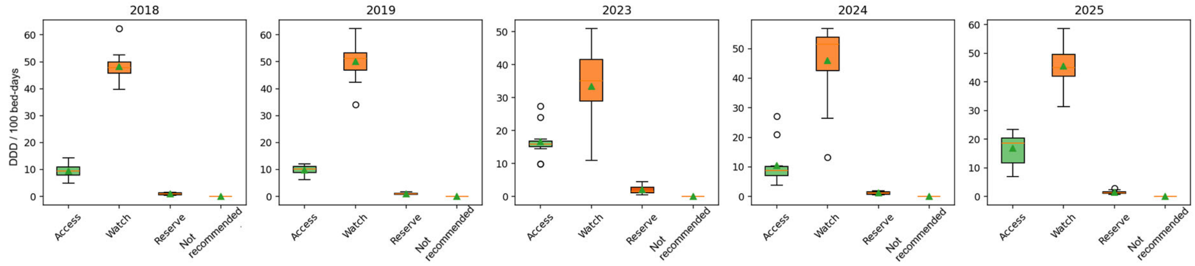

Figure S4. The distribution of antibiotic consumption by AWaRe categories by year at Sf. Ioan Hospital. The boxplots show the median (orange line), mean (green triangle), interquartile range (box), and outliers (open circles).

Table S3. Pairwise post hoc comparison of overall antibiotic consumption across pharmacological classes.

Group 1	Group 2	n1	n2	Statistic Test[a]	p-value	Adjusted p-value (p.adj)[b]	Significance Level (p.adj.signif)
Aminoglycosides	Carbapenems	360	720	-6.00	p<0.0001	p<0.0001	****
Aminoglycosides	Cephalosporins	360	2364	-13.58	p<0.0001	p<0.0001	****
Aminoglycosides	Cyclic lipopeptides	360	102	-10.52	p<0.0001	p<0.0001	****
Aminoglycosides	Fluoroquinolones	360	1080	-4.26	p<0.0001	p<0.01	**
Aminoglycosides	Glycopeptide	360	360	-3.08	0.0021	0.2824	ns
Aminoglycosides	Imidazole derivatives	360	360	4.29	p<0.0001	p<0.01	**
Aminoglycosides	Lincosamides	360	180	-8.07	p<0.0001	p<0.0001	****
Aminoglycosides	Macrolides	360	590	-14.71	p<0.0001	p<0.0001	****
Aminoglycosides	Monobactams	360	255	-13.85	p<0.0001	p<0.0001	****
Aminoglycosides	Oxizolidone	360	304	-4.89	p<0.0001	p<0.001	***
Aminoglycosides	Penicillins	360	1680	-6.67	p<0.0001	p<0.0001	****
Aminoglycosides	Phosphonic acid derivate	360	93	-6.45	p<0.0001	p<0.0001	****
Aminoglycosides	Polymyxins	360	180	4.47	p<0.0001	p<0.01	**
Aminoglycosides	Rifamycin	360	180	-4.19	p<0.0001	p<0.01	**
Aminoglycosides	Sulfonamides	360	165	-0.11	0.9111	1.0000	ns
Aminoglycosides	Tetracyclines	360	540	-9.24	p<0.0001	p<0.0001	****
Carbapenems	Cephalosporins	720	2364	-8.94	p<0.0001	p<0.0001	****
Carbapenems	Cyclic lipopeptides	720	102	-7.50	p<0.0001	p<0.0001	****
Carbapenems	Fluoroquinolones	720	1080	2.67	0.0077	1.0000	ns
Carbapenems	Glycopeptide	720	360	2.45	0.0144	1.0000	ns
Carbapenems	Imidazole derivatives	720	360	10.95	p<0.0001	p<0.0001	****
Carbapenems	Lincosamides	720	180	-4.20	p<0.0001	p<0.01	**
Carbapenems	Macrolides	720	590	-10.74	p<0.0001	p<0.0001	****
Carbapenems	Monobactams	720	255	-10.24	p<0.0001	p<0.0001	****
Carbapenems	Oxizolidone	720	304	0.10	0.9205	1.0000	ns
Carbapenems	Penicillins	720	1680	0.00	0.9966	1.0000	ns
Carbapenems	Phosphonic acid derivate	720	93	-3.29	0.0010	0.1363	ns
Carbapenems	Polymyxins	720	180	9.55	p<0.0001	p<0.0001	****
Carbapenems	Rifamycin	720	180	0.05	0.9573	1.0000	ns
Carbapenems	Sulfonamides	720	165	4.37	p<0.0001	p<0.01	**
Carbapenems	Tetracyclines	720	540	-4.24	p<0.0001	p<0.01	**
Cephalosporins	Cyclic lipopeptides	2364	102	-4.08	p<0.0001	p<0.01	**
Cephalosporins	Fluoroquinolones	2364	1080	13.86	p<0.0001	p<0.0001	****
Cephalosporins	Glycopeptide	2364	360	9.52	p<0.0001	p<0.0001	****

Cephalosporins	Imidazole derivatives	2364	360	19.23	p<0.0001	p<0.0001	****
Cephalosporins	Lincosamides	2364	180	0.40	0.6882	1.0000	ns
Cephalosporins	Macrolides	2364	590	-4.69	p<0.0001	p<0.001	***
Cephalosporins	Monobactams	2364	255	-5.54	p<0.0001	p<0.0001	****
Cephalosporins	Oxizolidone	2364	304	6.36	p<0.0001	p<0.0001	****
Cephalosporins	Penicillins	2364	1680	11.92	p<0.0001	p<0.0001	****
Cephalosporins	Phosphonic acid derivate	2364	93	0.17	0.8637	1.0000	ns
Cephalosporins	Polymyxins	2364	180	15.22	p<0.0001	p<0.0001	****
Cephalosporins	Rifamycin	2364	180	4.98	p<0.0001	p<0.0001	****
Cephalosporins	Sulfonamides	2364	165	9.41	p<0.0001	p<0.0001	****
Cephalosporins	Tetracyclines	2364	540	2.92	0.0035	0.4733	ns
Cyclic lipopeptides	Fluoroquinolones	102	1080	8.89	p<0.0001	p<0.0001	****
Cyclic lipopeptides	Glycopeptide	102	360	8.48	p<0.0001	p<0.0001	****
Cyclic lipopeptides	Imidazole derivatives	102	360	13.37	p<0.0001	p<0.0001	****
Cyclic lipopeptides	Lincosamides	102	180	3.58	0.0003	p<0.05	*
Cyclic lipopeptides	Macrolides	102	590	1.83	0.0668	1.0000	ns
Cyclic lipopeptides	Monobactams	102	255	0.40	0.6883	1.0000	ns
Cyclic lipopeptides	Oxizolidone	102	304	6.99	p<0.0001	p<0.0001	****
Cyclic lipopeptides	Penicillins	102	1680	7.77	p<0.0001	p<0.0001	****
Cyclic lipopeptides	Phosphonic acid derivate	102	93	3.00	0.0027	0.3638	ns
Cyclic lipopeptides	Polymyxins	102	180	12.82	p<0.0001	p<0.0001	****
Cyclic lipopeptides	Rifamycin	102	180	6.43	p<0.0001	p<0.0001	****
Cyclic lipopeptides	Sulfonamides	102	165	9.29	p<0.0001	p<0.0001	****
Cyclic lipopeptides	Tetracyclines	102	540	5.11	p<0.0001	p<0.0001	****
Fluoroquinolones	Glycopeptide	1080	360	0.49	0.6260	1.0000	ns
Fluoroquinolones	Imidazole derivatives	1080	360	9.51	p<0.0001	p<0.0001	****
Fluoroquinolones	Lincosamides	1080	180	-5.94	p<0.0001	p<0.0001	****
Fluoroquinolones	Macrolides	1080	590	-14.16	p<0.0001	p<0.0001	****
Fluoroquinolones	Monobactams	1080	255	-12.56	p<0.0001	p<0.0001	****
Fluoroquinolones	Oxizolidone	1080	304	-1.87	0.0615	1.0000	ns
Fluoroquinolones	Penicillins	1080	1680	-3.29	0.0010	0.1348	ns
Fluoroquinolones	Phosphonic acid derivate	1080	93	-4.54	p<0.0001	p<0.001	***
Fluoroquinolones	Polymyxins	1080	180	8.29	p<0.0001	p<0.0001	****
Fluoroquinolones	Rifamycin	1080	180	-1.54	0.1242	1.0000	ns
Fluoroquinolones	Sulfonamides	1080	165	2.97	0.0029	0.3985	ns
Fluoroquinolones	Tetracyclines	1080	540	-7.01	p<0.0001	p<0.0001	****
Glycopeptides	Imidazole derivatives	360	360	7.37	p<0.0001	p<0.0001	****
Glycopeptides	Lincosamides	360	180	-5.56	p<0.0001	p<0.0001	****
Glycopeptides	Macrolides	360	590	-11.28	p<0.0001	p<0.0001	****
Glycopeptides	Monobactams	360	255	-11.04	p<0.0001	p<0.0001	****

Glycopeptides	Oxizolidone	360	304	-1.94	0.0524	1.0000	ns
Glycopeptides	Penicillins	360	1680	-2.72	0.0065	0.8820	ns
Glycopeptides	Phosphonic acid derivates	360	93	-4.47	p<0.0001	p<0.01	**
Glycopeptides	Polymyxins	360	180	6.99	p<0.0001	p<0.0001	****
Glycopeptides	Rifamycin	360	180	-1.68	0.0928	1.0000	ns
Glycopeptides	Sulfonamides	360	165	2.33	0.0198	1.0000	ns
Glycopeptide	Tetracyclines	360	540	-5.87	p<0.0001	p<0.0001	****
Imidazole derivatives	Lincosamides	360	180	-11.58	p<0.0001	p<0.0001	****
Imidazole derivatives	Macrolides	360	590	-19.49	p<0.0001	p<0.0001	****
Imidazole derivatives	Monobactams	360	255	-17.75	p<0.0001	p<0.0001	****
Imidazole derivatives	Oxizolidone	360	304	-8.99	p<0.0001	p<0.0001	****
Imidazole derivatives	Penicillins	360	1680	-12.18	p<0.0001	p<0.0001	****
Imidazole derivatives	Phosphonic acid derivates	360	93	-9.20	p<0.0001	p<0.0001	****
Imidazole derivatives	Polymyxins	360	180	0.97	0.3312	1.0000	ns
Imidazole derivatives	Rifamycin	360	180	-7.70	p<0.0001	p<0.0001	****
Imidazole derivatives	Sulfonamides	360	165	-3.51	0.0004	0.0604	ns
Imidazole derivatives	Tetracyclines	360	540	-13.94	p<0.0001	p<0.0001	****
Lincosamides	Macrolides	180	590	-2.90	0.0037	0.5087	ns
Lincosamides	Monobactams	180	255	-4.07	p<0.0001	p<0.01	**
Lincosamides	Oxizolidone	180	304	3.79	0.0002	p<0.05	*
Lincosamides	Penicillins	180	1680	4.46	p<0.0001	p<0.01	**
Lincosamides	Phosphonic acid derivates	180	93	-0.10	0.9197	1.0000	ns
Lincosamides	Polymyxins	180	180	10.87	p<0.0001	p<0.0001	****
Lincosamides	Rifamycin	180	180	3.36	0.0008	0.1063	ns
Lincosamides	Sulfonamides	180	165	6.74	p<0.0001	p<0.0001	****
Lincosamides	Tetracyclines	180	540	1.26	0.2081	1.0000	ns
Macrolides	Monobactams	590	255	-2.00	0.0460	1.0000	ns
Macrolides	Oxizolidone	590	304	8.55	p<0.0001	p<0.0001	****
Macrolides	Penicillins	590	1680	12.46	p<0.0001	p<0.0001	****
Macrolides	Phosphonic acid derivates	590	93	2.10	0.0360	1.0000	ns
Macrolides	Polymyxins	590	180	16.35	p<0.0001	p<0.0001	****
Macrolides	Rifamycin	590	180	7.06	p<0.0001	p<0.0001	****
Macrolides	Sulfonamides	590	165	11.05	p<0.0001	p<0.0001	****
Macrolides	Tetracyclines	590	540	5.96	p<0.0001	p<0.0001	****

Monobactams	Oxizolidone	255	304	8.87	p<0.0001	p<0.0001	****
Monobactams	Penicillins	255	1680	11.10	p<0.0001	p<0.0001	****
Monobactams	Phosphonic acid derivates	255	93	3.17	0.0015	0.2101	ns
Monobactams	Polymyxins	255	180	15.84	p<0.0001	p<0.0001	****
Monobactams	Rifamycin	255	180	7.71	p<0.0001	p<0.0001	****
Monobactams	Sulfonamides	255	165	11.24	p<0.0001	p<0.0001	****
Monobactams	Tetracyclines	255	540	6.64	p<0.0001	p<0.0001	****
Oxizolidone	Penicillins	304	1680	-0.11	0.9104	1.0000	ns
Oxizolidone	Phosphonic acid derivates	304	93	-3.12	0.0018	0.2487	ns
Oxizolidone	Polymyxins	304	180	8.39	p<0.0001	p<0.0001	****
Oxizolidone	Rifamycin	304	180	-0.03	0.9800	1.0000	ns
Oxizolidone	Sulfonamides	304	165	3.83	0.0001	p<0.05	*
Oxizolidone	Tetracyclines	304	540	-3.46	0.0005	0.0733	ns
Penicillins	Phosphonic acid derivates	1680	93	-3.40	0.0007	0.0913	ns
Penicillins	Polymyxins	1680	180	10.15	p<0.0001	p<0.0001	****
Penicillins	Rifamycin	1680	180	0.06	0.9527	1.0000	ns
Penicillins	Sulfonamides	1680	165	4.62	p<0.0001	p<0.001	***
Penicillins	Tetracyclines	1680	540	-4.87	p<0.0001	p<0.001	***
Phosphonic acid derivates	Polymyxins	93	180	9.07	p<0.0001	p<0.0001	****
Phosphonic acid derivates	Rifamycin	93	180	2.87	0.0041	0.5520	ns
Phosphonic acid derivates	Sulfonamides	93	165	5.70	p<0.0001	p<0.0001	****
Phosphonic acid derivates	Tetracyclines	93	540	1.08	0.2803	1.0000	ns
Polymyxins	Rifamycin	180	180	-7.51	p<0.0001	p<0.0001	****
Polymyxins	Sulfonamides	180	165	-3.89	0.0001	p<0.05	*
Polymyxins	Tetracyclines	180	540	-12.05	p<0.0001	p<0.0001	****
Rifamycin	Sulfonamides	180	165	3.46	0.0005	0.0747	ns
Rifamycin	Tetracyclines	180	540	-2.86	0.0043	0.5844	ns
Sulfonamides	Tetracyclines	165	540	-6.95	p<0.0001	p<0.0001	****

n1, n2= sample sizes for analysed groups; [a] post hoc pairwise comparisons were performed using the Dunn test; [b] Adjusted p-values for multiple testing using Bonferroni correction method; significance level: * p < 0.05, ** p <0.01, *** p <0.001, **** p < 0.0001, ns= no significant difference (p ≥ 0.05); antibiotic consumption was expressed as DDD / 100 bed-days.

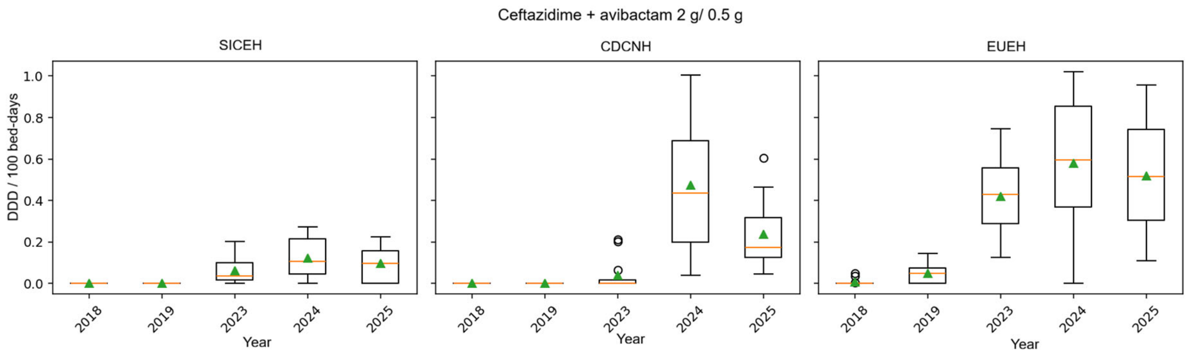

SICEH= Sf. Ioan Clinical Emergency Hospital; CDCNH= Dr. Carol Davila Clinical Nephrology Hospital; EUEH= Elias University Emergency Hospital; DDD = Defined Daily Dose.

Figure S5. The distribution of Ceftazidime/avibactam consumption across hospitals and study years.
The boxplots show the median (orange line), mean (green triangle), interquartile range (box) and outliers (open circles).

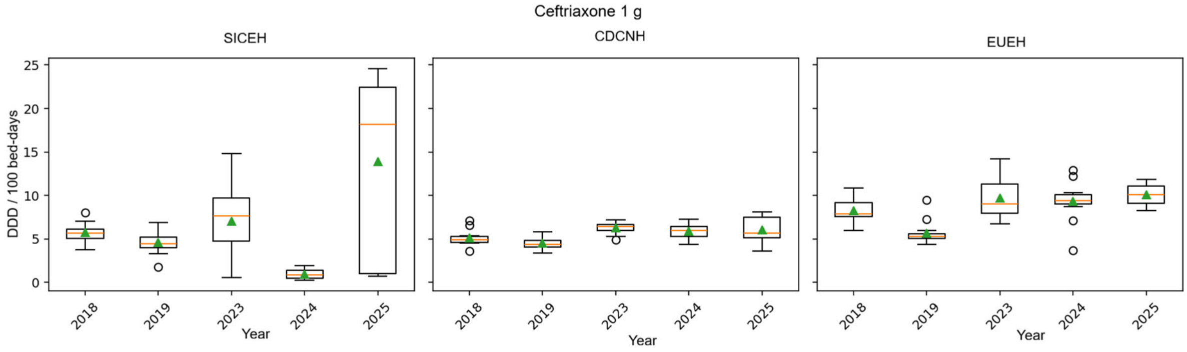

SICEH= Sf. Ioan Clinical Emergency Hospital; CDCNH= Dr. Carol Davila Clinical Nephrology Hospital; EUEH= Elias University Emergency Hospital; DDD = Defined Daily Dose.

Figure S6. The distribution of Ceftriaxone consumption across hospitals and study years. The boxplots show the median (orange line), mean (green triangle), interquartile range (box), and outliers (open circles).

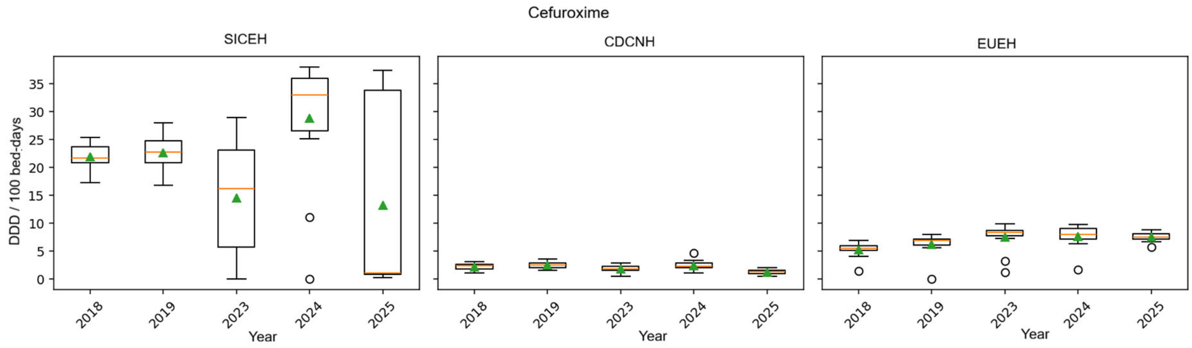

**Cefuroxime consumption represents the combined usage of both oral (750 mg) and parenteral (1.5 g) formulations, expressed in DDD/ 100 bed-days; SICEH= Sf. Ioan Clinical Emergency Hospital; CDCNH= Dr. Carol Davila Clinical Nephrology Hospital; EUEH= Elias University Emergency Hospital; DDD = Defined Daily Dose.*

Figure S7. The distribution of Cefuroxime consumption across hospitals and study years.
The boxplots show the median (orange line), mean (green triangle), interquartile range (box), and outliers (open circles).

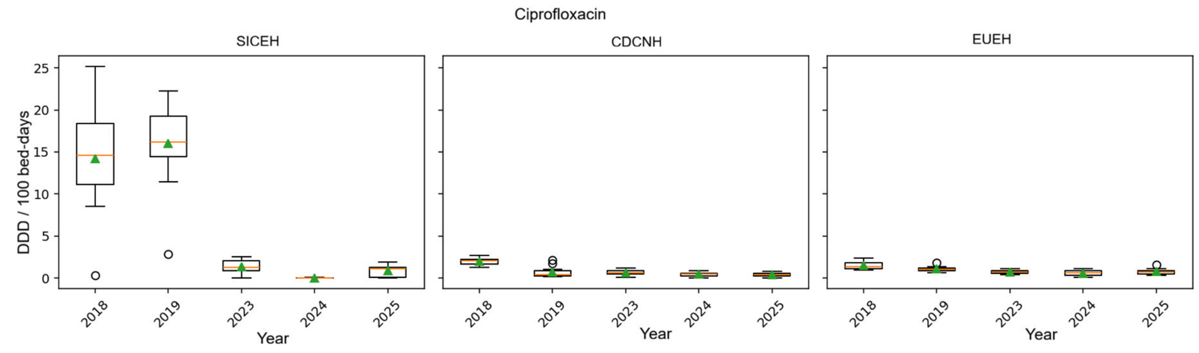

**Ciprofloxacin consumption represents the combined usage of both oral (500 mg) and parenteral (100mg / mL) formulations, expressed in DDD/ 100 bed-days; SICEH= Sf. Ioan Clinical Emergency Hospital; CDCNH= Dr. Carol Davila Clinical Nephrology Hospital; EUEH= Elias University Emergency Hospital; DDD = Defined Daily Dose.*

Figure S8. The distribution of Ciprofloxacin consumption across hospitals and study years.
The boxplots show the median (orange line), mean (green triangle), interquartile range (box), and outliers (open circles).

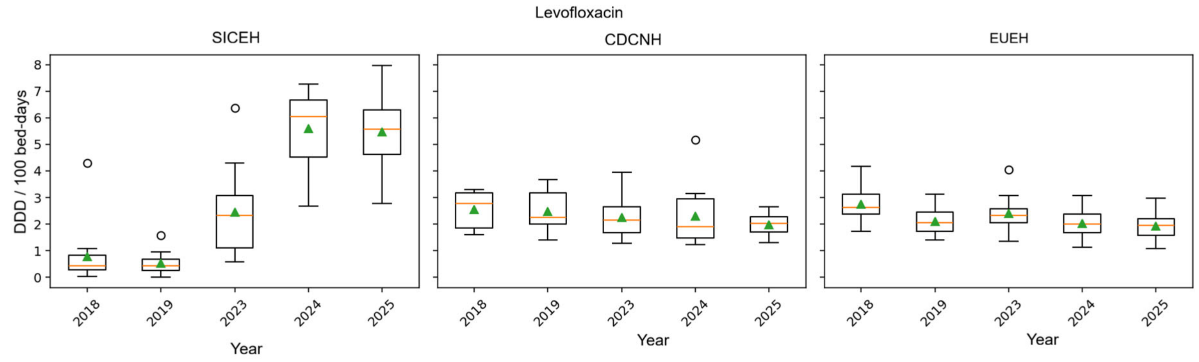

**Levofloxacin consumption represents the combined usage of both oral (500 mg) and parenteral (500 mg / mL) formulations, expressed in DDD/ 100 bed-days; SICEH= Sf. Ioan Clinical Emergency Hospital; CDCNH= Dr. Carol Davila Clinical Nephrology Hospital; EUEH= Elias University Emergency Hospital; DDD = Defined Daily Dose.*

Figure S9. The distribution of Levofloxacin consumption across hospitals and study years.
The boxplots show the median (orange line), mean (green triangle), interquartile range (box), and outliers (open circles).

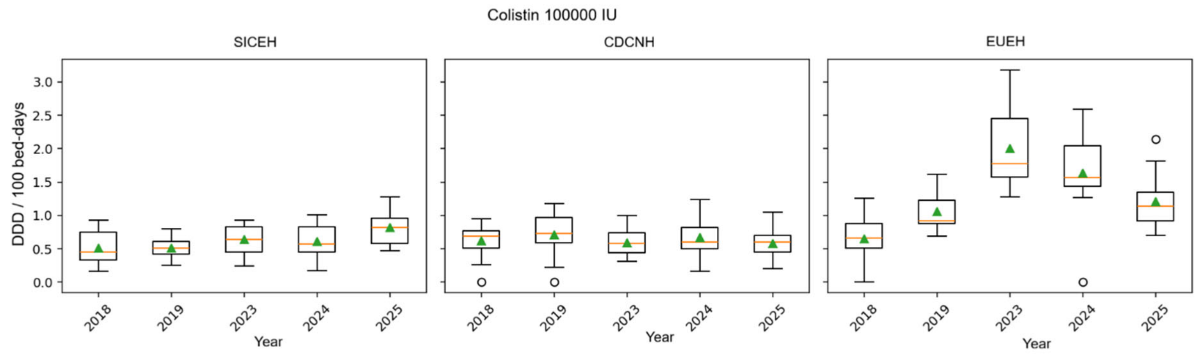

SICEH= Sf. Ioan Clinical Emergency Hospital; CDCNH= Dr. Carol Davila Clinical Nephrology Hospital; EUEH= Elias University Emergency Hospital; DDD = Defined Daily Dose; IU= international unit.

Figure S10. The distribution of Colistin consumption across hospitals and study years.
The boxplots show the median (orange line), mean (green triangle), interquartile range (box), and outliers (open circles).

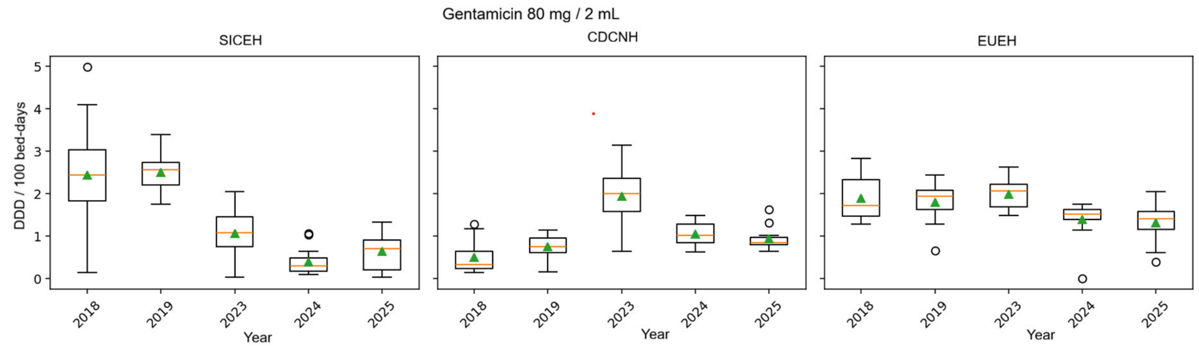

SICEH= Sf. Ioan Clinical Emergency Hospital; CDCNH= Dr. Carol Davila Clinical Nephrology Hospital; EUEH= Elias University Emergency Hospital; DDD = Defined Daily Dose.

Figure S11. The distribution of Gentamicin consumption across hospitals and study years.
The boxplots show the median (orange line), mean (green triangle), interquartile range (box), and outliers (open circles).

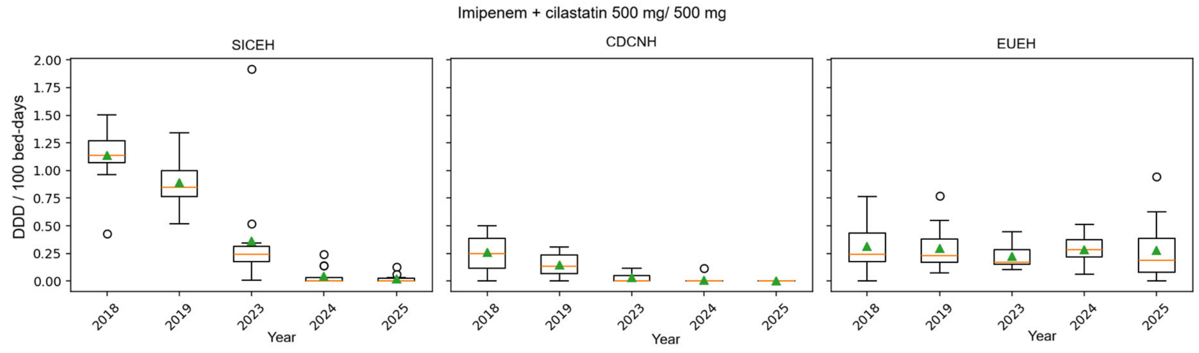

SICEH= Sf. Ioan Clinical Emergency Hospital; CDCNH= Dr. Carol Davila Clinical Nephrology Hospital; EUEH= Elias University Emergency Hospital; DDD = Defined Daily Dose.

Figure S12. The distribution of Imipenem + cilastatin consumption across hospitals and study years.
The boxplots show the median (orange line), mean (green triangle), interquartile range (box), and outliers (open circles).

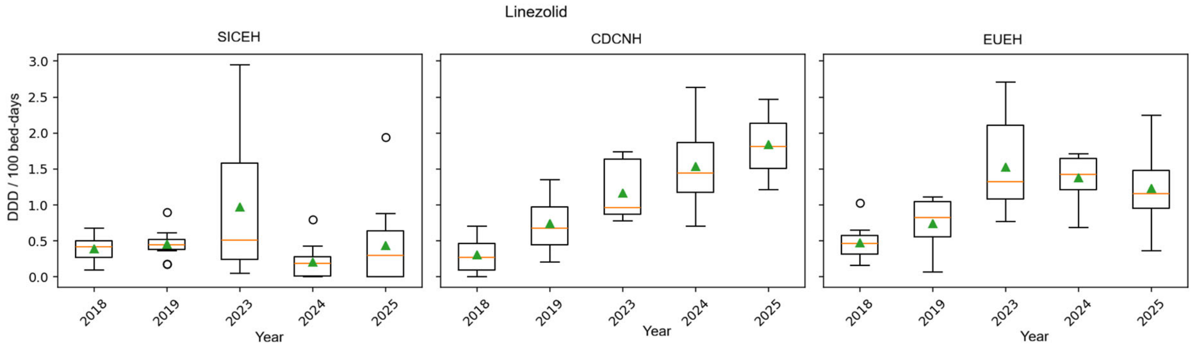

**Linezolid consumption represents the combined usage of both oral (600 mg) and parenteral (2 mg/ mL) formulations, expressed in DDD/ 100 bed-days; SICEH= Sf. Ioan Clinical Emergency Hospital; CDCNH= Dr. Carol Davila Clinical Nephrology Hospital; EUEH= Elias University Emergency Hospital; DDD = Defined Daily Dose.*

Figure S13. The distribution of Linezolid consumption across hospitals and study years.
The boxplots show the median (orange line), mean (green triangle), interquartile range (box), and outliers (open circles).

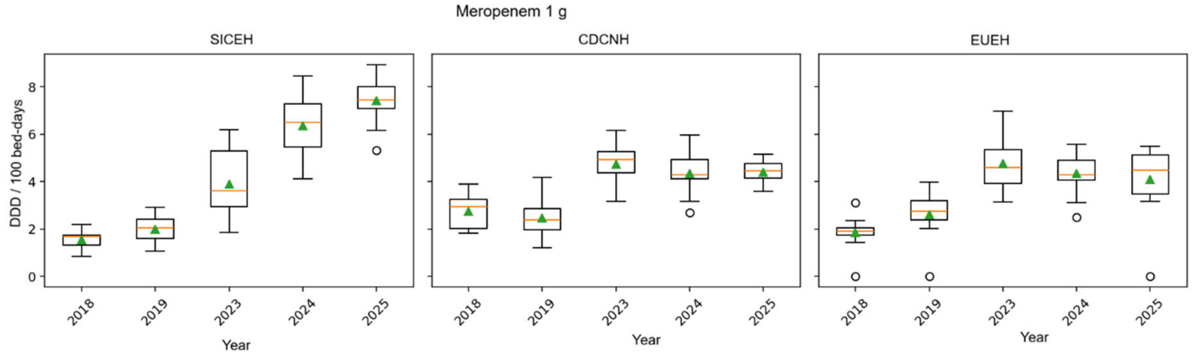

SICEH= Sf. Ioan Clinical Emergency Hospital; CDCNH= Dr. Carol Davila Clinical Nephrology Hospital; EUEH= Elias University Emergency Hospital; DDD = Defined Daily Dose.

Figure S14. The distribution of Meropenem consumption across hospitals and study years.
The boxplots show the median (orange line), mean (green triangle), interquartile range (box), and outliers (open circles).

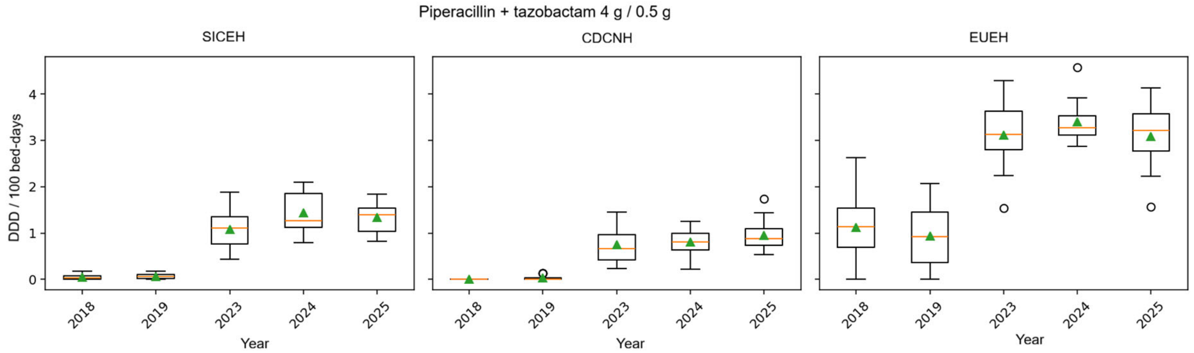

SICEH= Sf. Ioan Clinical Emergency Hospital; CDCNH= Dr. Carol Davila Clinical Nephrology Hospital; EUEH= Elias University Emergency Hospital; DDD = Defined Daily Dose.

Figure S15. The distribution of Piperacillin + tazobactam consumption across hospitals and study years.
The boxplots show the median (orange line), mean (green triangle), interquartile range (box), and outliers (open circles).

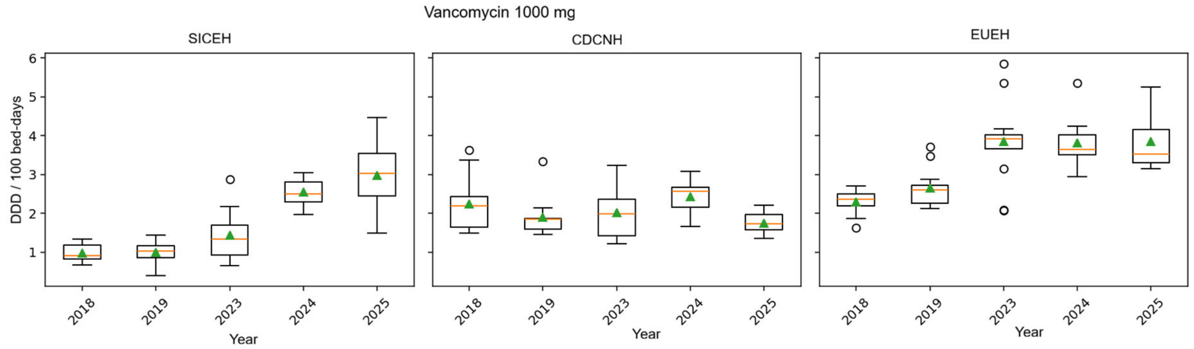

SICEH= Sf. Ioan Clinical Emergency Hospital; CDCNH= Dr. Carol Davila Clinical Nephrology Hospital; EUEH= Elias University Emergency Hospital; DDD = Defined Daily Dose.

Figure S16. The distribution of Vancomycin consumption across hospitals and study years.
The boxplots show the median (orange line), mean (green triangle), interquartile range (box), and outliers (open circles).

Table S4. Pairwise post hoc comparison of 12 selected antibiotic consumption across study years within each hospital.

Antibiotic	Hospital	Year 1	Year 2	p-value[a]	Adjusted p-value (p_holm)	Significance
Ceftazidine+Avibactam 2 g/ 0,5 g	SICEH	2018	2023	0.0004	0.0031	Yes
Ceftazidine+Avibactam 2 g/ 0,5 g	SICEH	2018	2024	0.0000	0.0004	Yes
Ceftazidine+Avibactam 2 g/ 0,5 g	SICEH	2018	2025	0.0011	0.0065	Yes
Ceftazidine+Avibactam 2 g/ 0,5 g	SICEH	2019	2023	0.0004	0.0031	Yes
Ceftazidine+Avibactam 2 g/ 0,5 g	SICEH	2019	2024	0.0000	0.0004	Yes
Ceftazidine+Avibactam 2 g/ 0,5 g	SICEH	2019	2025	0.0011	0.0065	Yes

Ceftazidine+Avibactam 2 g/ 0,5 g	CDCNH	2018	2024	0.0000	0.0001	Yes
Ceftazidine+Avibactam 2 g/ 0,5 g	CDCNH	2018	2025	0.0000	0.0001	Yes
Ceftazidine+Avibactam 2 g/ 0,5 g	CDCNH	2019	2024	0.0000	0.0001	Yes
Ceftazidine+Avibactam 2 g/ 0,5 g	CDCNH	2019	2025	0.0000	0.0001	Yes
Ceftazidine+Avibactam 2 g/ 0,5 g	CDCNH	2023	2024	0.0002	0.0013	Yes
Ceftazidine+Avibactam 2 g/ 0,5 g	CDCNH	2023	2025	0.0006	0.0032	Yes
Ceftazidine+Avibactam 2 g/ 0,5 g	EUEH	2018	2023	0.0000	0.0002	Yes
Ceftazidine+Avibactam 2 g/ 0,5 g	EUEH	2018	2024	0.0006	0.0036	Yes
Ceftazidine+Avibactam 2 g/ 0,5 g	EUEH	2018	2025	0.0000	0.0002	Yes
Ceftazidine+Avibactam 2 g/ 0,5 g	EUEH	2019	2023	0.0001	0.0004	Yes
Ceftazidine+Avibactam 2 g/ 0,5 g	EUEH	2019	2024	0.0027	0.0137	Yes
Ceftazidine+Avibactam 2 g/ 0,5 g	EUEH	2019	2025	0.0000	0.0004	Yes
Ceftriaxone 1 g	SICEH	2018	2024	0.0000	0.0004	Yes
Ceftriaxone 1 g	SICEH	2019	2024	0.0000	0.0004	Yes
Ceftriaxone 1 g	SICEH	2023	2024	0.0017	0.0132	Yes
Ceftriaxone 1 g	CDCNH	2018	2023	0.0061	0.0427	Yes
Ceftriaxone 1 g	CDCNH	2019	2023	0.0001	0.0012	Yes
Ceftriaxone 1 g	CDCNH	2019	2024	0.0009	0.0081	Yes
Ceftriaxone 1 g	CDCNH	2019	2025	0.0051	0.0409	Yes
Ceftriaxone 1 g	EUEH	2018	2019	0.0006	0.0047	Yes
Ceftriaxone 1 g	EUEH	2018	2025	0.0043	0.0256	Yes
Ceftriaxone 1 g	EUEH	2019	2023	0.0003	0.0028	Yes
Ceftriaxone 1 g	EUEH	2019	2024	0.0020	0.0141	Yes
Ceftriaxone 1 g	EUEH	2019	2025	0.0002	0.0016	Yes
Cefuroxime	SICEH	2023	2024	0.0035	0.0355	Yes
Cefuroxime	CDCNH	2018	2025	0.0011	0.0088	Yes
Cefuroxime	CDCNH	2019	2025	0.0005	0.0043	Yes
Cefuroxime	CDCNH	2024	2025	0.0003	0.0031	Yes
Cefuroxime	EUEH	2018	2023	0.0051	0.0409	Yes
Cefuroxime	EUEH	2018	2024	0.0007	0.0066	Yes
Cefuroxime	EUEH	2018	2025	0.0002	0.0020	Yes
Ciprofloxacin	SICEH	2018	2023	0.0004	0.0019	Yes
Ciprofloxacin	SICEH	2018	2024	0.0000	0.0004	Yes
Ciprofloxacin	SICEH	2018	2025	0.0002	0.0015	Yes
Ciprofloxacin	SICEH	2019	2023	0.0000	0.0004	Yes
Ciprofloxacin	SICEH	2019	2024	0.0000	0.0004	Yes
Ciprofloxacin	SICEH	2019	2025	0.0000	0.0004	Yes
Ciprofloxacin	SICEH	2023	2024	0.0042	0.0167	Yes
Ciprofloxacin	CDCNH	2018	2019	0.0007	0.0051	Yes
Ciprofloxacin	CDCNH	2018	2023	0.0000	0.0004	Yes
Ciprofloxacin	CDCNH	2018	2024	0.0000	0.0004	Yes
Ciprofloxacin	CDCNH	2018	2025	0.0000	0.0004	Yes
Ciprofloxacin	EUEH	2018	2023	0.0001	0.0010	Yes
Ciprofloxacin	EUEH	2018	2024	0.0001	0.0010	Yes
Ciprofloxacin	EUEH	2018	2025	0.0004	0.0031	Yes
Ciprofloxacin	EUEH	2019	2023	0.0061	0.0366	Yes

Ciprofloxacin	EUEH	2019	2024	0.0043	0.0299	Yes
Colistin 100000 IU	SICEH	2019	2025	0.0029	0.0295	Yes
Colistin 100000 IU	EUEH	2018	2019	0.0102	0.0407	Yes
Colistin 100000 IU	EUEH	2018	2023	0.0000	0.0004	Yes
Colistin 100000 IU	EUEH	2018	2024	0.0005	0.0038	Yes
Colistin 100000 IU	EUEH	2018	2025	0.0029	0.0176	Yes
Colistin 100000 IU	EUEH	2019	2023	0.0002	0.0014	Yes
Colistin 100000 IU	EUEH	2019	2024	0.0043	0.0213	Yes
Colistin 100000 IU	EUEH	2023	2025	0.0017	0.0116	Yes
Colistin 100000 IU	EUEH	2024	2025	0.0141	0.0424	Yes
Gentamicin 80 mg/ 2 ml	SICEH	2018	2023	0.0073	0.0363	Yes
Gentamicin 80 mg/ 2 ml	SICEH	2018	2024	0.0007	0.0051	Yes
Gentamicin 80 mg/ 2 ml	SICEH	2018	2025	0.0020	0.0121	Yes
Gentamicin 80 mg/ 2 ml	SICEH	2019	2023	0.0001	0.0008	Yes
Gentamicin 80 mg/ 2 ml	SICEH	2019	2024	0.0000	0.0004	Yes
Gentamicin 80 mg/ 2 ml	SICEH	2019	2025	0.0000	0.0004	Yes
Gentamicin 80 mg/ 2 ml	SICEH	2023	2024	0.0073	0.0363	Yes
Gentamicin 80 mg/ 2 ml	CDCNH	2018	2023	0.0002	0.0016	Yes
Gentamicin 80 mg/ 2 ml	CDCNH	2018	2024	0.0020	0.0141	Yes
Gentamicin 80 mg/ 2 ml	CDCNH	2018	2025	0.0043	0.0213	Yes
Gentamicin 80 mg/ 2 ml	CDCNH	2019	2023	0.0002	0.0022	Yes
Gentamicin 80 mg/ 2 ml	CDCNH	2023	2024	0.0024	0.0146	Yes
Gentamicin 80 mg/ 2 ml	CDCNH	2023	2025	0.0014	0.0108	Yes
Gentamicin 80 mg/ 2 ml	EUEH	2023	2024	0.0029	0.0265	Yes
Gentamicin 80 mg/ 2 ml	EUEH	2023	2025	0.0011	0.0111	Yes
Imipenem + Cilastatin 500 mg/ 500 mg	SICEH	2018	2019	0.0120	0.0240	Yes
Imipenem + Cilastatin 500 mg/ 500 mg	SICEH	2018	2023	0.0007	0.0029	Yes
Imipenem + Cilastatin 500 mg/ 500 mg	SICEH	2018	2024	0.0000	0.0002	Yes
Imipenem + Cilastatin 500 mg/ 500 mg	SICEH	2018	2025	0.0000	0.0003	Yes
Imipenem + Cilastatin 500 mg/ 500 mg	SICEH	2019	2023	0.0007	0.0029	Yes
Imipenem + Cilastatin 500 mg/ 500 mg	SICEH	2019	2024	0.0000	0.0002	Yes
Imipenem + Cilastatin 500 mg/ 500 mg	SICEH	2019	2025	0.0000	0.0003	Yes
Imipenem + Cilastatin 500 mg/ 500 mg	SICEH	2023	2024	0.0004	0.0021	Yes
Imipenem + Cilastatin 500 mg/ 500 mg	SICEH	2023	2025	0.0003	0.0017	Yes
Imipenem + Cilastatin 500 mg/ 500 mg	CDCNH	2018	2023	0.0008	0.0047	Yes
Imipenem + Cilastatin 500 mg/ 500 mg	CDCNH	2018	2024	0.0001	0.0008	Yes
Imipenem + Cilastatin 500 mg/ 500 mg	CDCNH	2018	2025	0.0000	0.0004	Yes
Imipenem + Cilastatin 500 mg/ 500 mg	CDCNH	2019	2023	0.0055	0.0275	Yes
Imipenem + Cilastatin 500 mg/ 500 mg	CDCNH	2019	2024	0.0002	0.0012	Yes
Imipenem + Cilastatin 500 mg/ 500 mg	CDCNH	2019	2025	0.0000	0.0004	Yes
Imipenem + Cilastatin 500 mg/ 500 mg	CDCNH	2023	2025	0.0071	0.0282	Yes
Levofloxacin	SICEH	2018	2023	0.0020	0.0060	Yes
Levofloxacin	SICEH	2018	2024	0.0001	0.0006	Yes
Levofloxacin	SICEH	2018	2025	0.0001	0.0006	Yes
Levofloxacin	SICEH	2019	2023	0.0006	0.0036	Yes
Levofloxacin	SICEH	2019	2024	0.0000	0.0004	Yes

Levofloxacin	SICEH	2019	2025	0.0000	0.0004	Yes
Levofloxacin	SICEH	2023	2024	0.0007	0.0037	Yes
Levofloxacin	SICEH	2023	2025	0.0009	0.0037	Yes
Linezolid	CDCNH	2018	2019	0.0051	0.0204	Yes
Linezolid	CDCNH	2018	2023	0.0000	0.0004	Yes
Linezolid	CDCNH	2018	2024	0.0000	0.0004	Yes
Linezolid	CDCNH	2018	2025	0.0000	0.0004	Yes
Linezolid	CDCNH	2019	2023	0.0166	0.0497	Yes
Linezolid	CDCNH	2019	2024	0.0014	0.0081	Yes
Linezolid	CDCNH	2019	2025	0.0001	0.0004	Yes
Linezolid	CDCNH	2023	2025	0.0014	0.0081	Yes
Linezolid	EUEH	2018	2023	0.0001	0.0007	Yes
Linezolid	EUEH	2018	2024	0.0001	0.0006	Yes
Linezolid	EUEH	2018	2025	0.0004	0.0031	Yes
Linezolid	EUEH	2019	2023	0.0020	0.0121	Yes
Linezolid	EUEH	2019	2024	0.0006	0.0041	Yes
Linezolid	EUEH	2019	2025	0.0073	0.0363	Yes
Meropenem 1 g	SICEH	2018	2023	0.0001	0.0005	Yes
Meropenem 1 g	SICEH	2018	2024	0.0000	0.0004	Yes
Meropenem 1 g	SICEH	2018	2025	0.0000	0.0004	Yes
Meropenem 1 g	SICEH	2019	2023	0.0020	0.0060	Yes
Meropenem 1 g	SICEH	2019	2024	0.0000	0.0004	Yes
Meropenem 1 g	SICEH	2019	2025	0.0000	0.0004	Yes
Meropenem 1 g	SICEH	2023	2024	0.0014	0.0054	Yes
Meropenem 1 g	SICEH	2023	2025	0.0001	0.0005	Yes
Meropenem 1 g	CDCNH	2018	2023	0.0003	0.0018	Yes
Meropenem 1 g	CDCNH	2018	2024	0.0005	0.0024	Yes
Meropenem 1 g	CDCNH	2018	2025	0.0001	0.0008	Yes
Meropenem 1 g	CDCNH	2019	2023	0.0001	0.0008	Yes
Meropenem 1 g	CDCNH	2019	2024	0.0002	0.0014	Yes
Meropenem 1 g	CDCNH	2019	2025	0.0001	0.0008	Yes
Meropenem 1 g	EUEH	2018	2019	0.0047	0.0186	Yes
Meropenem 1 g	EUEH	2018	2023	0.0000	0.0004	Yes
Meropenem 1 g	EUEH	2018	2024	0.0000	0.0004	Yes
Meropenem 1 g	EUEH	2018	2025	0.0005	0.0037	Yes
Meropenem 1 g	EUEH	2019	2023	0.0002	0.0012	Yes
Meropenem 1 g	EUEH	2019	2024	0.0007	0.0044	Yes
Meropenem 1 g	EUEH	2019	2025	0.0022	0.0110	Yes
Piperacillin + Tazobactam 4 g/ 0.5 g	SICEH	2018	2023	0.0000	0.0004	Yes
Piperacillin + Tazobactam 4 g/ 0.5 g	SICEH	2018	2024	0.0000	0.0004	Yes
Piperacillin + Tazobactam 4 g/ 0.5 g	SICEH	2018	2025	0.0000	0.0004	Yes
Piperacillin + Tazobactam 4 g/ 0.5 g	SICEH	2019	2023	0.0000	0.0004	Yes
Piperacillin + Tazobactam 4 g/ 0.5 g	SICEH	2019	2024	0.0000	0.0004	Yes
Piperacillin + Tazobactam 4 g/ 0.5 g	SICEH	2019	2025	0.0000	0.0004	Yes
Piperacillin + Tazobactam 4 g/ 0.5 g	CDCNH	2018	2023	0.0000	0.0001	Yes
Piperacillin + Tazobactam 4 g/ 0.5 g	CDCNH	2018	2024	0.0000	0.0001	Yes

Piperacillin + Tazobactam 4 g/ 0.5 g	CDCNH	2018	2025	0.0000	0.0001	Yes
Piperacillin + Tazobactam 4 g/ 0.5 g	CDCNH	2019	2023	0.0000	0.0002	Yes
Piperacillin + Tazobactam 4 g/ 0.5 g	CDCNH	2019	2024	0.0000	0.0002	Yes
Piperacillin + Tazobactam 4 g/ 0.5 g	CDCNH	2019	2025	0.0000	0.0002	Yes
Piperacillin + Tazobactam 4 g/ 0.5 g	EUEH	2018	2023	0.0002	0.0008	Yes
Piperacillin + Tazobactam 4 g/ 0.5 g	EUEH	2018	2024	0.0000	0.0004	Yes
Piperacillin + Tazobactam 4 g/ 0.5 g	EUEH	2018	2025	0.0001	0.0006	Yes
Piperacillin + Tazobactam 4 g/ 0.5 g	EUEH	2019	2023	0.0001	0.0006	Yes
Piperacillin + Tazobactam 4 g/ 0.5 g	EUEH	2019	2024	0.0000	0.0004	Yes
Piperacillin + Tazobactam 4 g/ 0.5 g	EUEH	2019	2025	0.0001	0.0006	Yes
Vancomycin 1000 mg	SICEH	2018	2024	0.0000	0.0004	Yes
Vancomycin 1000 mg	SICEH	2018	2025	0.0000	0.0004	Yes
Vancomycin 1000 mg	SICEH	2019	2024	0.0000	0.0004	Yes
Vancomycin 1000 mg	SICEH	2019	2025	0.0000	0.0004	Yes
Vancomycin 1000 mg	SICEH	2023	2024	0.0006	0.0030	Yes
Vancomycin 1000 mg	SICEH	2023	2025	0.0005	0.0029	Yes
Vancomycin 1000 mg	CDCNH	2024	2025	0.0011	0.0111	Yes
Vancomycin 1000 mg	EUEH	2018	2023	0.0029	0.0177	Yes
Vancomycin 1000 mg	EUEH	2018	2024	0.0000	0.0004	Yes
Vancomycin 1000 mg	EUEH	2018	2025	0.0000	0.0004	Yes
Vancomycin 1000 mg	EUEH	2019	2023	0.0086	0.0431	Yes
Vancomycin 1000 mg	EUEH	2019	2024	0.0002	0.0020	Yes
Vancomycin 1000 mg	EUEH	2019	2025	0.0005	0.0033	Yes

SICEH= Sf. Ioan Clinical Emergency Hospital; CDCNH= Dr. Carol Davila Clinical Nephrology Hospital; EUEH= Elias University Emergency Hospital; [a] post hoc pairwise comparisons were performed using the Mann–Whitney U test; [b] Adjusted p-values for multiple testing with the Holm correction method;
cefuroxime consumption represents the combined usage of both oral (750 mg) and parenteral (1.5 g) formulations; ciprofloxacin consumption represents the combined usage of both oral (500 mg) and parenteral (100mg / mL) formulations; levofloxacin consumption represents the combined usage of both oral (500 mg) and parenteral (500 mg / mL) formulations; linezolid consumption represents the combined usage of both oral (600 mg) and parenteral (2 mg/ mL) formulations; IU= international unit; antibiotic consumption was expressed as DDD / 100 bed-days.
*Only statistically significant pairwise comparisons were presented in the table (adjusted p-value <0.05); non-significant comparisons were omitted to optimize space.

Table S5. Pairwise post hoc comparison of 12 selected antibiotic consumption across hospitals for each study year.

Antibiotic	Year	Hospital1	Hospital2	p-value[a]	Adjusted p-value (p_holm)	Significance
Ceftazidine+Avibactam 2 g/ 0,5 g	2019	SICEH	EUEH	0.0011	0.0033	Yes
Ceftazidine+Avibactam 2 g/ 0,5 g	2019	CDCNH	EUEH	0.0011	0.0033	Yes
Ceftazidine+Avibactam 2 g/ 0,5 g	2023	SICEH	EUEH	p<0.0001	0.0002	Yes
Ceftazidine+Avibactam 2 g/ 0,5 g	2023	CDCNH	EUEH	p<0.0001	0.0002	Yes
Ceftazidine+Avibactam 2 g/ 0,5 g	2024	SICEH	CDCNH	0.0086	0.0172	Yes
Ceftazidine+Avibactam 2 g/ 0,5 g	2024	SICEH	EUEH	0.0051	0.0152	Yes
Ceftazidine+Avibactam 2 g/ 0,5 g	2025	SICEH	CDCNH	0.0459	0.0459	Yes

Ceftazidine+Avibactam 2 g/ 0,5 g	2025	SICEH	EUEH	0.0006	0.0017	Yes
Ceftazidine+Avibactam 2 g/ 0,5 g	2025	CDCNH	EUEH	0.0166	0.0331	Yes
Ceftriaxone 1 g	2018	SICEH	EUEH	0.0005	0.0010	Yes
Ceftriaxone 1 g	2018	CDCNH	EUEH	p<0.0001	0.0003	Yes
Ceftriaxone 1 g	2019	CDCNH	EUEH	0.0073	0.0218	Yes
Ceftriaxone 1 g	2023	CDCNH	EUEH	p<0.0001	0.0002	Yes
Ceftriaxone 1 g	2024	SICEH	CDCNH	p<0.0001	0.0001	Yes
Ceftriaxone 1 g	2024	SICEH	EUEH	p<0.0001	0.0001	Yes
Ceftriaxone 1 g	2024	CDCNH	EUEH	0.0009	0.0009	Yes
Ceftriaxone 1 g	2025	CDCNH	EUEH	p<0.0001	0.0001	Yes
Cefuroxime	2018	SICEH	CDCNH	p<0.0001	0.0001	Yes
Cefuroxime	2018	SICEH	EUEH	p<0.0001	0.0001	Yes
Cefuroxime	2018	CDCNH	EUEH	0.0004	0.0004	Yes
Cefuroxime	2019	SICEH	CDCNH	p<0.0001	0.0001	Yes
Cefuroxime	2019	SICEH	EUEH	p<0.0001	0.0001	Yes
Cefuroxime	2019	CDCNH	EUEH	0.0006	0.0006	Yes
Cefuroxime	2023	CDCNH	EUEH	0.0004	0.0012	Yes
Cefuroxime	2024	SICEH	CDCNH	0.0006	0.0014	Yes
Cefuroxime	2024	SICEH	EUEH	0.0006	0.0014	Yes
Cefuroxime	2024	CDCNH	EUEH	0.0005	0.0014	Yes
Cefuroxime	2025	CDCNH	EUEH	p<0.0001	0.0001	Yes
Ciprofloxacin	2018	SICEH	CDCNH	0.0006	0.0018	Yes
Ciprofloxacin	2018	SICEH	EUEH	0.0006	0.0018	Yes
Ciprofloxacin	2018	CDCNH	EUEH	0.0304	0.0304	Yes
Ciprofloxacin	2019	SICEH	CDCNH	p<0.0001	0.0001	Yes
Ciprofloxacin	2019	SICEH	EUEH	p<0.0001	0.0001	Yes
Ciprofloxacin	2019	CDCNH	EUEH	0.0262	0.0262	Yes
Ciprofloxacin	2024	SICEH	CDCNH	p<0.0001	0.0001	Yes
Ciprofloxacin	2024	SICEH	EUEH	p<0.0001	0.0001	Yes
Ciprofloxacin	2025	CDCNH	EUEH	0.0073	0.0218	Yes
Colistin 100000 IU	2019	SICEH	EUEH	p<0.0001	0.0002	Yes
Colistin 100000 IU	2019	CDCNH	EUEH	0.0226	0.0452	Yes
Colistin 100000 IU	2023	SICEH	EUEH	p<0.0001	0.0001	Yes
Colistin 100000 IU	2023	CDCNH	EUEH	p<0.0001	0.0001	Yes
Colistin 100000 IU	2024	SICEH	EUEH	0.0006	0.0018	Yes
Colistin 100000 IU	2024	CDCNH	EUEH	0.0006	0.0018	Yes
Colistin 100000 IU	2025	SICEH	CDCNH	0.0351	0.0351	Yes
Colistin 100000 IU	2025	SICEH	EUEH	0.0166	0.0331	Yes
Colistin 100000 IU	2025	CDCNH	EUEH	0.0002	0.0007	Yes
Gentamicin 80 mg / 2 mL	2018	SICEH	CDCNH	0.0017	0.0033	Yes
Gentamicin 80 mg / 2 mL	2018	CDCNH	EUEH	p<0.0001	0.0001	Yes
Gentamicin 80 mg / 2 mL	2019	SICEH	CDCNH	p<0.0001	0.0001	Yes
Gentamicin 80 mg / 2 mL	2019	SICEH	EUEH	0.0024	0.0024	Yes
Gentamicin 80 mg / 2 mL	2019	CDCNH	EUEH	0.0002	0.0003	Yes
Gentamicin 80 mg / 2 mL	2023	SICEH	CDCNH	0.0051	0.0102	Yes
Gentamicin 80 mg / 2 mL	2023	SICEH	EUEH	0.0007	0.0022	Yes

Gentamicin 80 mg / 2 mL	2024	SICEH	CDCNH	0.0007	0.0018	Yes
Gentamicin 80 mg / 2 mL	2024	SICEH	EUEH	0.0006	0.0018	Yes
Gentamicin 80 mg / 2 mL	2024	CDCNH	EUEH	0.0029	0.0029	Yes
Gentamicin 80 mg / 2 mL	2025	SICEH	EUEH	0.0024	0.0073	Yes
Imipenem + Cilastatin 500 mg/ 500 mg	2018	SICEH	CDCNH	p<0.0001	0.0002	Yes
Imipenem + Cilastatin 500 mg/ 500 mg	2018	SICEH	EUEH	p<0.0001	0.0002	Yes
Imipenem + Cilastatin 500 mg/ 500 mg	2019	SICEH	CDCNH	p<0.0001	0.0001	Yes
Imipenem + Cilastatin 500 mg/ 500 mg	2019	SICEH	EUEH	0.0001	0.0002	Yes
Imipenem + Cilastatin 500 mg/ 500 mg	2023	SICEH	CDCNH	0.0004	0.0009	Yes
Imipenem + Cilastatin 500 mg/ 500 mg	2023	CDCNH	EUEH	p<0.0001	0.0001	Yes
Imipenem + Cilastatin 500 mg/ 500 mg	2024	SICEH	EUEH	0.0002	0.0004	Yes
Imipenem + Cilastatin 500 mg/ 500 mg	2024	CDCNH	EUEH	p<0.0001	p<0.0001	Yes
Imipenem + Cilastatin 500 mg/ 500 mg	2025	SICEH	CDCNH	0.0071	0.0071	Yes
Imipenem + Cilastatin 500 mg/ 500 mg	2025	SICEH	EUEH	0.0008	0.0016	Yes
Imipenem + Cilastatin 500 mg/ 500 mg	2025	CDCNH	EUEH	p<0.0001	0.0001	Yes
Levofloxacin	2018	SICEH	CDCNH	0.0006	0.0018	Yes
Levofloxacin	2018	SICEH	EUEH	0.0006	0.0018	Yes
Levofloxacin	2019	SICEH	CDCNH	p<0.0001	0.0002	Yes
Levofloxacin	2019	SICEH	EUEH	p<0.0001	0.0002	Yes
Levofloxacin	2024	SICEH	CDCNH	0.0002	0.0004	Yes
Levofloxacin	2024	SICEH	EUEH	p<0.0001	0.0001	Yes
Levofloxacin	2025	SICEH	CDCNH	p<0.0001	0.0001	Yes
Levofloxacin	2025	SICEH	EUEH	p<0.0001	0.0001	Yes
Linezolid	2024	SICEH	CDCNH	p<0.0001	0.0001	Yes
Linezolid	2024	SICEH	EUEH	p<0.0001	0.0001	Yes
Linezolid	2025	SICEH	CDCNH	0.0002	0.0007	Yes
Linezolid	2025	SICEH	EUEH	0.0011	0.0022	Yes
Linezolid	2025	CDCNH	EUEH	0.0029	0.0029	Yes
Meropenem 1 g	2018	SICEH	CDCNH	0.0002	0.0006	Yes
Meropenem 1 g	2018	CDCNH	EUEH	0.0120	0.0240	Yes
Meropenem 1 g	2024	SICEH	CDCNH	0.0020	0.0060	Yes
Meropenem 1 g	2024	SICEH	EUEH	0.0020	0.0060	Yes
Meropenem 1 g	2025	SICEH	CDCNH	p<0.0001	0.0001	Yes
Meropenem 1 g	2025	SICEH	EUEH	p<0.0001	0.0001	Yes
Piperacillin + Tazobactam 4 g/ 0.5 g	2018	SICEH	CDCNH	0.0011	0.0011	Yes
Piperacillin + Tazobactam 4 g/ 0.5 g	2018	SICEH	EUEH	0.0004	0.0007	Yes
Piperacillin + Tazobactam 4 g/ 0.5 g	2018	CDCNH	EUEH	p<0.0001	0.0001	Yes
Piperacillin + Tazobactam 4 g/ 0.5 g	2019	CDCNH	EUEH	0.0025	0.0075	Yes
Piperacillin + Tazobactam 4 g/ 0.5 g	2023	SICEH	EUEH	p<0.0001	0.0001	Yes
Piperacillin + Tazobactam 4 g/ 0.5 g	2023	CDCNH	EUEH	p<0.0001	0.0001	Yes
Piperacillin + Tazobactam 4 g/ 0.5 g	2024	SICEH	CDCNH	0.0020	0.0020	Yes
Piperacillin + Tazobactam 4 g/ 0.5 g	2024	SICEH	EUEH	p<0.0001	0.0001	Yes
Piperacillin + Tazobactam 4 g/ 0.5 g	2024	CDCNH	EUEH	p<0.0001	0.0001	Yes
Piperacillin + Tazobactam 4 g/ 0.5 g	2025	SICEH	CDCNH	0.0141	0.0141	Yes
Piperacillin + Tazobactam 4 g/ 0.5 g	2025	SICEH	EUEH	p<0.0001	0.0002	Yes
Piperacillin + Tazobactam 4 g/ 0.5 g	2025	CDCNH	EUEH	p<0.0001	0.0001	Yes

Vancomycin 1000 mg	2018	SICEH	CDCNH	p<0.0001	0.0001	Yes
Vancomycin 1000 mg	2018	SICEH	EUEH	p<0.0001	0.0001	Yes
Vancomycin 1000 mg	2019	SICEH	CDCNH	p<0.0001	0.0001	Yes
Vancomycin 1000 mg	2019	SICEH	EUEH	p<0.0001	0.0001	Yes
Vancomycin 1000 mg	2019	CDCNH	EUEH	0.0005	0.0005	Yes
Vancomycin 1000 mg	2023	SICEH	CDCNH	0.0351	0.0351	Yes
Vancomycin 1000 mg	2023	SICEH	EUEH	p<0.0001	0.0003	Yes
Vancomycin 1000 mg	2023	CDCNH	EUEH	0.0007	0.0015	Yes
Vancomycin 1000 mg	2024	SICEH	EUEH	p<0.0001	0.0001	Yes
Vancomycin 1000 mg	2024	CDCNH	EUEH	p<0.0001	0.0001	Yes
Vancomycin 1000 mg	2025	SICEH	CDCNH	0.0005	0.0010	Yes
Vancomycin 1000 mg	2025	SICEH	EUEH	0.0226	0.0226	Yes
Vancomycin 1000 mg	2025	CDCNH	EUEH	p<0.0001	0.0001	Yes

SICEH= Sf. Ioan Clinical Emergency Hospital; CDCNH= Dr. Carol Davila Clinical Nephrology Hospital; EUEH= Elias University Emergency Hospital; [a] post hoc pairwise comparisons were performed using the Mann–Whitney U test; [b] Adjusted p-values for multiple testing with the Holm correction method;
cefuroxime consumption represents the combined usage of both oral and parenteral (1.5 g) formulations; ciprofloxacin consumption represents the combined usage of both oral (500 mg) and parenteral (100mg / mL) formulations; levofloxacin consumption represents the combined usage of both oral (500 mg) and parenteral (500 mg / mL) formulations; linezolid consumption represents the combined usage of both oral (600 mg) and parenteral (2 mg/ mL) formulations; IU= international unit; antibiotic consumption was expressed as DDD / 100 bed-days.
*Only statistically significant pairwise comparisons were presented in the table (adjusted p-value <0.05); non-significant comparisons were omitted to optimize space.

Table S6. Annual antibiotic consumption by pharmacological class and selected substances across hospitals.

Therapeutic Class / Antibiotic	DDD / 100 bed-days														
	SICEH					EUMH					CDCNH				
	**2018**	**2019**	**2023**	**2024**	**2025**	**2018**	**2019**	**2023**	**2024**	**2025**	**2018**	**2019**	**2023**	**2024**	**2025**
**Cephalosporins**	350.35	345.37	343.54	425.12	420.43	182.5	163.39	227.85	236.92	237.17	100.56	96.89	111.62	117.77	98.58
Cefuroxime	264	272.52	174.97	345.95	159.72	63.86	74.85	90.75	92.37	90.85	26.52	29.89	22.14	29.67	15.19
Ceftriaxone 1 g	69.03	55	84.51	11.53	166.78	99.41	68.47	116.31	112.04	121.09	61.52	54.07	75.73	71.25	72.59
Ceftazidime/Avibactam 2 g/ 0,5 g	0	0	0.75	1.48	1.18	0.09	0.58	5.03	6.96	6.23	0	0	0.48	5.67	2.85
**Carbapenems**	32.94	39.41	52.99	77.74	90.21	30.1	39.84	65.52	60.95	55.99	36.04	31.36	57.16	52.33	53.61
Meropenem 1 g	18.45	23.75	46.79	76.39	88.94	22.19	31.4	57.06	51.97	48.95	32.94	29.45	56.66	52.19	52.86
Imipenem/Cilastatin 500 mg/ 500 mg	13.68	10.69	4.37	0.52	0.26	3.8	3.53	2.67	3.4	3.37	3.11	1.78	0.37	0.11	0
**Fluoroquinolones**	181.01	198.42	47.98	68.08	76.17	68.6	46.83	40.19	36.74	35.77	56.33	39.98	48.01	37.61	37.54
Ciprofloxacin	170.97	191.95	16.14	0.3	10.65	18.39	13.14	8.88	7.33	9.28	23.92	8.69	8.06	5.61	5.13
Levofloxacin	9.5	6.33	29.35	67.23	65.53	33.13	25.36	28.76	24.35	23.19	30.49	29.82	27.08	27.72	23.88
**Penicillins**	15.11	15.64	46.73	25.91	24.31	43.39	67.84	104.96	127.16	126.63	57.87	39.16	55.62	74.25	70.04
Piperacillin/Tazobactam 4 g/ 0.5 g	0.65	0.79	13.03	17.18	16.12	13.54	11.15	37.35	40.86	37.07	0	0.39	8.93	9.69	11.46
**Glycopeptides**	12.62	12.53	17.62	30.59	36.15	27.71	31.78	46.39	46.1	46.76	27.01	22.75	24.37	29.17	21.08
Vancomycin 1000 mg	11.87	11.96	17.26	30.59	35.78	27.6	31.78	46.16	45.76	46.16	27.01	22.75	24.2	29.17	21.08
**Oxizolidones**	4.7	5.42	11.65	2.46	5.25	5.64	8.85	18.33	16.52	14.81	3.69	8.87	13.96	18.47	22.13
Linezolid	4.7	5.42	11.65	2.46	5.25	5.64	8.85	18.33	16.52	14.81	3.69	8.87	13.96	18.47	22.13
**Aminoglycosides**	30.72	31.26	13.61	5.23	7.91	23.33	22.66	24.2	17.03	16.32	6.13	9.16	24.67	13.92	12.56
Gentamicin 80 mg / 2 mL	29.23	30.14	12.87	4.83	7.76	22.77	21.72	23.89	16.7	15.85	6.08	8.98	23.27	12.6	11.26
**Polymyxins**	6.11	6.14	7.65	7.33	9.78	7.79	12.75	24.07	19.6	14.46	7.39	8.48	7.09	8.08	6.99
Colistin 100000 IU	6.11	6.14	7.65	7.33	9.78	7.79	12.75	24.07	19.6	14.46	7.39	8.48	7.09	8.08	6.99
**Phosphonic acid derivate**	0.00	0.00	0.00	0.00	0.00	0.21	0.28	0.08	1.03	0.95	0.00	0.00	1.28	0.73	1.51
Fosfomycin 40 mg / mL	0.00	0.00	0.00	0.00	0.00	0.21	0.28	0.08	1.03	0.95	0.00	0.00	1.28	0.73	1.51
**Rifamycin**	0.00	3.17	4.21	0.00	0.00	0.00	0.00	7.53	6.48	7.05	10.53	17.82	44.24	35.66	26.29

Rifamixin 200 mg	0.00	3.17	4.21	0.00	0.00	0.00	0.00	7.53	6.48	7.05	10.53	17.82	44.24	35.66	26.29
**Sulfonamides**	0.00	0.93	2.19	2.06	7.11	6.34	6.97	5.03	6.11	8.46	1.74	5.31	5.29	6.61	5.43
Sulfamethoxazol + Trimethoprim 400 mg / 80 mg	0.00	0.93	2.19	2.06	7.11	6.34	6.97	5.03	6.11	8.46	1.74	5.31	5.29	6.61	5.43
**Tetracyclines**	0.94	0.94	8.46	3.66	22.75	3.45	7.65	12.84	24.02	16.02	6.34	19.30	15.17	11.61	11.73
Tigecycline 50 mg	0.02	0.64	4.72	2.20	0.00	2.75	5.12	6.66	14.47	9.73	4.53	10.74	4.58	4.13	2.66
Eravacicline 100 mg	0.25	0.30	0.00	0.00	0.00	0.69	0.00	0.520	0.00	0.572	0.00	0.00	0.00	0.20	0.44
**Macrolides**	0.59	1.01	2.10	0.10	0.29	0.86	2.37	6.29	11.74	12.48	3.03	2.05	4.27	7.93	11.06
Azithromycin 500 mg	0.00	0.97	2.04	0.00	0.18	0.00	0.00	3.25	4.80	9.28	0.00	0.00	1.50	3.24	4.21
Clarithromycin 500 mg	0.59	0.04	0.06	0.10	0.12	0.86	2.37	3.04	6.95	3.20	3.03	2.05	2.77	4.69	6.85
**Lincosamides**	0.44	0.50	0.09	0.11	0.05	0.00	0.00	0.13	0.60	1.35	1.79	4.51	7.36	6.87	6.09
Clindamycin 300 mg	0.44	0.50	0.09	0.11	0.05	0.00	0.00	0.13	0.60	1.35	1.79	4.51	7.36	6.87	6.09
**Imidazole derivatives**	66.05	69.54	66.54	42.58	63.01	8.28	5.66	6.15	6.19	7.15	23.10	20.71	27.22	32.02	46.44
Metronidazole 250 mg	11.91	7.94	5.04	8.76	9.61	3.66	2.50	2.44	2.45	2.49	5.35	2.51	1.37	1.77	4.17
Metronidazole 5 mg / mL	54.14	61.60	61.50	33.81	53.41	4.63	3.16	3.71	3.73	4.66	17.75	18.20	25.86	30.25	42.27

SICEH= Sf. Ioan Clinical Emergency Hospital; CDCNH= Dr. Carol Davila Clinical Nephrology Hospital; EUEH= Elias University Emergency Hospital; cefuroxime consumption represents the combined usage of both oral and parenteral formulations; ciprofloxacin consumption represents the combined usage of both oral (500 mg) and parenteral (100mg / mL) formulations; levofloxacin consumption represents the combined usage of both oral (500 mg) and parenteral (500 mg / mL) formulations; linezolid consumption represents the combined usage of both oral (600 mg) and parenteral (2 mg/ mL) formulations.
